# cGAS/STING and innate brain inflammation following acute high-fat feeding

**DOI:** 10.3389/fimmu.2022.1012594

**Published:** 2022-09-29

**Authors:** Sarah E. Elzinga, Rosemary Henn, Benjamin J. Murdock, Bhumsoo Kim, John M. Hayes, Faye Mendelson, Ian Webber-Davis, Sam Teener, Crystal Pacut, Stephen I. Lentz, Eva L. Feldman

**Affiliations:** ^1^ Department of Neurology, University of Michigan, Ann Arbor, MI, United States; ^2^ NeuroNetwork for Emerging Therapies, University of Michigan, Ann Arbor, MI, United States; ^3^ Department of Internal Medicine, Division of Metabolism, Endocrinology and Diabetes, University of Michigan, Ann Arbor, MI, United States

**Keywords:** cGAS/STING, acute, innate inflammation, microglia, high fat diet

## Abstract

Obesity, prediabetes, and diabetes are growing in prevalence worldwide. These metabolic disorders are associated with neurodegenerative diseases, particularly Alzheimer’s disease and Alzheimer’s disease related dementias. Innate inflammatory signaling plays a critical role in this association, potentially *via* the early activation of the cGAS/STING pathway. To determine acute systemic metabolic and inflammatory responses and corresponding changes in the brain, we used a high fat diet fed obese mouse model of prediabetes and cognitive impairment. We observed acute systemic changes in metabolic and inflammatory responses, with impaired glucose tolerance, insulin resistance, and alterations in peripheral immune cell populations. Central inflammatory changes included microglial activation in a pro-inflammatory environment with cGAS/STING activation. Blocking gap junctions in neuron-microglial co-cultures significantly decreased cGAS/STING activation. Collectively these studies suggest a role for early activation of the innate immune system both peripherally and centrally with potential inflammatory crosstalk between neurons and glia.

## Introduction

Global incidences of obesity, prediabetes, and diabetes are increasing worldwide (1, 2). Obesity rates have burgeoned in recent years, growing to pandemic proportions (3). Global diabetes rates topped 463 million in 2019, with an estimated additional 374 million people having impaired glucose tolerance and prediabetes (1). This alarming rise in the rates of obesity and metabolic disease predispose individuals to complications of the central nervous system (CNS), including mild cognitive impairment, Alzheimer’s disease or Alzheimer’s disease related dementias (AD/ADRD) (4–6).

Chronic inflammation and immune system dysregulation are common in individuals with obesity and in individuals who fall along the continuum of metabolic dysfunction from prediabetes to frank type 2 diabetes (7). Previous studies investigating the effects of metabolic dysfunction on the CNS report dysregulation of immune and inflammatory mechanisms, typically increased glial activation and elevated production of CNS pro-inflammatory proteins and mediators (8–10). Specifically, a high-fat diet (HFD) in mice induces an inflammatory phenotype in microglia, the resident immune cells of the CNS (11, 12). Additionally, HFD or other CNS pro-inflammatory events increase trafficking of peripheral immune cells into the brain (13–15), further promoting neuroinflammation.

Although evidence supports a role for CNS inflammation in obesity, prediabetes, and diabetes, previous studies primarily focus on later disease time points, and few have investigated how HFD-induced obesity and prediabetes impact short-term inflammatory changes. Innate inflammatory pathways with an acute response to damage or danger signals may potentially respond to metabolic stress to mediate early CNS responses to HFD. In a dysmetabolic environment, elevated fatty acids can activate innate inflammatory mechanisms and upregulate pro-inflammatory cytokine production (16, 17). This in turn up-regulates downstream feed-forward mechanisms, such as signaling through the interferon-α receptor (18), which further contributes to a pro-inflammatory environment.

One innate inflammatory pathway implicated in the cellular response to metabolic dysfunction is the cGAS/STING (cyclic GMP-AMP/stimulator of interferon genes) pathway (19–21). cGAS/STING is a cytosolic double-stranded DNA (dsDNA) sensing pathway, which responds to viral or bacterial dsDNA as well as self dsDNA, *e.g.*, from damaged nuclei or mitochondria *via* cGAS and working through its adaptor molecule STING and transcription factors interferon regulatory factor 3 (IRF3) and nuclear factor kappa beta (NFkB) to upregulate pro-inflammatory gene expression. In the periphery or peripheral cells, HFD or treatment with the saturated fatty acid palmitate upregulates cGAS/STING signaling (22). cGAS/STING also contributes to pro-inflammatory feed forward mechanisms *via* inflammatory crosstalk between neighboring cells *via* gap junctions (23). Further, cGAS/STING is implicated in the pathology of CNS neurodegenerative diseases, such as AD/ADRD (24–26), Parkinson’s disease (27), and amyotrophic lateral sclerosis (28), and may thus constitute a “bridge” between metabolic dysfunction and cognitive impairment.

In the current study, we examined CNS activation of the cGAS/STING pathway in mice fed a high fat diet (HFD) for 3 d. We focused our studies on the primary immune cells of the brain, microglia, capable of inflammatory crosstalk with neurons *via* gap junctions (23). We leveraged our HFD mouse model, which develops obesity and prediabetes along with cognitive impairment upon acute and chronic feeding (29). We observed systemic changes in metabolic and inflammatory responses, with impaired glucose tolerance, insulin resistance, and alterations in peripheral immune cell populations after just 3 d of HFD. We also identified central inflammatory changes, with microglial and cGAS/STING pathway activation. Additionally, in our neuron-microglial co-culture system, reducing cell to cell inflammatory crosstalk by blocking gap junctions significantly reduced cGAS/STING activation. These findings support an early role for cGAS/STING in response to HFD *via* neuron-glial inflammatory crosstalk and suggest a pivotal role for acute activation of innate immune mechanisms in the CNS in response to global metabolic dysfunction.

## Materials and methods

### Experimental animals and study design

Four-wk-old C57BL/6J male mice obtained from The Jackson Laboratory (catalog # 000664; Bar Harbor, ME). Animals were housed with no more than five littermates per cage in a pathogen free room at 20 ± 2 °C with a 12 h light/dark cycle at the University of Michigan Unit for Laboratory Animal Medicine and monitored daily by veterinary staff. Animals were provided food and water *ad libitum* and a minimum of one enrichment item (nestlet and/or enviropak). Following a 1 or 2 wk acclimation period, animals were assigned randomly to their respective diets (Research Diets, New Brunswick, NJ) as follows: standard diet (SD; 10% calories from fat; catalog # D12450J) or high-fat diet (HFD; 60% calories from fat; catalog # D12492). A subset of animals were used for cognitive phenotyping (see puzzle box), which was performed on day 2 of diet and for a duration of 3d. Animals were sacrificed (detailed below) on the final day of puzzle box (4 d on diet). For all other animals, after 3 d on diet mice underwent glucose tolerance testing (see metabolic phenotyping) and were sacrificed (detailed below) the next day (4 d on diet). Four hours prior to euthanasia, animals were fasted and a subset of animals within both the SD and HFD groups were given intraperitoneal injection of either saline (5 mL/kg body weight [BW]) or lipopolysaccharide (LPS; catalog # tlrl-3pelps, Invivogen, San Diego, CA) at a dose of 500 µg LPS/kg BW in total volume of 5 mL/kg BW saline. At terminal, animals were euthanized *via* intraperitoneal injection of 150 mg/kg pentobarbital (Fatal-Plus, Vortech Pharmaceuticals, Dearborn, MI). Blood was removed from the vena cava and animals were perfused with phosphate buffered saline prior to removal of tissues. Cortex tissue was used to determine *ex vivo* CNS insulin sensitivity using western blotting, plasma and hemi-brains for immunophenotyping using flow cytometry, plasma for inflammatory cytokines using ELISA, hemi-brains for microglial morphology using immunohistochemistry, and hippocampal tissue for cGAS/STING pathway protein expression using Western blotting (all methods detailed below). The University of Michigan’s Institutional Animal Care and Use Committee approved all animal protocols (PRO0010039).

### Metabolic phenotyping and immunophenotyping

Glucose tolerance testing (GTT) was performed after 3 d of diet as previously (30, 31). Briefly, 10% D-glucose at 1g glucose/1kg body weight was injected intraperitoneally after a 4 h fast and glucose measurements taken at baseline and 15-, 30-, 60-, and 120-min post injection. Blood glucose levels were determined from a tail blood sample using a glucometer (AlphaTRAK, Abbot Laboratories, Chicago, IL) and appropriate glucose strips (Zoetis, Parsippany, NJ).

After 4 d HFD feeding, immunophenotyping was performed on peripheral blood samples and on CNS tissue using flow cytometry (32) to determine circulating immune cell populations, as previously published (32, 33). Fluorescently labeled leukocytes were classified by staining with antibodies (Biolegend, San Diego, CA) for well-characterized surface markers (**Table 1**). Briefly, doublets were excluded using forward scatter width (FSC-W) and forward scatter height (FSC-H) where events farther than 10% from the diagonal were excluded. In both tissue types, lymphocytes were characterized as CD45+, SSC-low cells expressing CD3 and either CD4+ or CD8+, while NK cells were characterized as CD45+, SSC-low, CD3–, NK1.1+, and CD49b+. B cells in the periphery were characterized as CD45+, SSC-low, CD3-, and CD19+ and were not detectible in the CNS. Myeloid populations in the blood were characterized as CD45+ and CD11b+: neutrophils were Ly6G+ while monocytes were Ly6G– and either Ly6C– or Ly6C+. In the CNS, myeloid cells were CD45+, CD3–, CD19. Ly6G+ cells were identified as neutrophils, Ly6G-, CD11b+, CD45-high, and Ly6C+ were identified as Ly6C+ monocytes, and Ly6G-, CD11b+, CD45-mid cells were identified as microglia. In both tissue types, monocytes, microglia, and neutrophils were further assessed for F4/80 or CD11c surface expression by their median fluorescent intensity as a proxy for activation state. A FACSAria II (BD Biosciences, San Jose, CA) was used to run samples and FlowJo software (FlowJo, Ashland, OR) to analyze results.

**Table 1:** Flow cytometry antibodies for blood and CNS immune cell characterization.

	BV421	FITC	PE	PerCP-5.5	APC	PE-Cy7	APC-Cy7
**Lymphoid (Blood and CNS)**	CD8	CD3	Nk1.1	CD19	CD45	CD49b	CD4
CD4 T-cells	+	+	–	–	+	–	+
CD8 T-cells	+	+	–	–	+	–	–
NK cells	–	–	+	–	+	+	–
B cells (CNS; not detectible)	–	–	–	+	+	–	–
**Myeloid (Blood)**	Cd11c	Ly6c	F4/80	CD3/CD19	CD45	Ly6G	CD11b
Neutrophils	MFI	–	MFI	–	+	+	+
Ly6C- Monocytes	MFI	–	MFI	–	+	–	+
Ly6C+ Monocytes	MFI	+	MFI	–	+	–	+
**Myeloid (CNS)**							
Neutrophils	MFI	–	MFI	–	+	+	+
Ly6C+ Monocytes	MFI	+	MFI	–	+	–	+
Microglia	MFI	–	MFI	–	+/-	–	+

+/- (with/without).

### Microglial morphology

As previously (34), we performed analysis of microglial morphology for three regions of the hippocampus, the hilus, molecular layer, and CA1 regions. In brief, hemi-brains were dissected and fixed for 48 h in 4% paraformaldehyde. Following a sucrose gradient (10%, 20%, and 30% for 24 h each), hemi-brains were embedded in OCT and frozen at -80°C. Brains were sectioned (50 µm) and stained (rabbit anti-Iba1, 1:1000; catalog # 019-19741, Wako, Richmond, VA) in 6-well plates in floating tissue sections. Secondary antibody (anti-rabbit Alexa-fluor Plus 594, 1:2000; catalog # A32740, Invitrogen) and Hoechst nuclear stain were applied, and sections were mounted using ProLong Gold (Invitrogen). A Leica Stellaris 8 Falcon Confocal Microscope and a 40X oil immersion objective was used to take Z-stack images (30 µm). Images were processed with Imaris Software (Oxford Instruments) and open microscopy environment TIF files used to analyze microglial territorial volume, cell volume, percent occupied volume, average branch length, maximum branch length, minimum branch length, number of end points, and number of end points using a modified 3DMorph script in MATLAB (MathWorks, Natick, MA), as previously published (34).

### *Ex vivo* insulin stimulation

On day 4 of diet after sacrifice and perfusion, right cortex was dissected and placed in a 12-well plate containing media (Neruobasal, 5% pen-strep, MN additives (Sigma, St Louis, MO); 10 mg/mL bovine serum albumin, 10 mg/mL apo-transferrin, 0.1 mg/mL biotin, 15 mg/mL D-galactose, 0.63 mg/mL progesterone, 16 mg/mL putrescine, 50 μg/mL selenium, 50 μg/mL β-estradiol, 50 μg/mL hydrocortisone, 16 mg/mL catalase, 2.5 mg/mL SOD). Tissue was finely minced with scissors and split into two microcentrifuge tubes (one for unstimulated control and one for insulin) containing 300 μL media. Tubes were placed into an incubator (37°C, 5% CO_2_) for 30 min. Following the 30 min incubation, insulin (20 nM) or an equivalent volume of media was added to the appropriate tubes. Tubes were returned to the incubator for 45 min and inverted several times every 10-15 min. Following the 45 min incubation, tubes were spun down (1 min, 4°C, 17,000 g), media removed, and tissue snap frozen in liquid N_2_. Tissue was maintained at -80°C for later Western blot (WB) analysis.

### ELISA and WB

On day 4 of diet, blood was collected, and plasma isolated for inflammatory cytokine analysis *via* ELISA. ELISA was performed for TNF-α and MCP-1 by the University of Michigan Rogel Cancer Center Immunology Core. Cortex and hippocampal tissue as well as neuronal and microglia cells were homogenized in RIPA buffer (Pierce, Rockford, IL) with protease inhibitors (Roche Diagnostics, Indianapolis, IN), sonicated, and centrifuged (30 min, 4°C, 13,300 rpm) in preparation for WB, which was performed as previously published (35, 36). All samples were normalized for equal protein concentration prior to loading. Nitrocellulose membranes were blocked (Tris buffered saline [TBS], 0.01% Tween-20, 5% bovine serum albumin [BSA]) for 2 h, primary antibodies (varying concentrations in TBS, 0.01% Tween-20, 5% BSA) were incubated overnight at 4°C, and secondary antibodies (varying concentrations in TBS, 0.01% Tween-20, 5% milk) were incubated for 1.5 h at room temperature. SuperSignal West Femto Maximum Sensitivity Substrate (Pierce, Rockford, IL) or Clarity Max (Biorad, Hercules, CA) was used to visualize signal and images were captured by a ChemiDoc (Biorad) or with x-ray film. Images were analyzed using ImageJ (37) or Image Lab software (Biorad). Insulin signaling primary antibodies were: pAkt (catalog # 4060), Akt (catalog # 4691), pIRS-1 (pSer307, catalog # 2381; pSer636/639, catalog # 2388), IRS-1 (catalog # 3407), all from Cell Signaling Technologies (Danvers, MA) and diluted at 1:1000. cGAS/STING pathway primary antibodies (Cell Signaling Technologies) were: cGAS (catalog # 31659S; 1:1000), STING (catalog # 50494S; 1:1000), pIRF3 (S396; catalog # 4947S; 1:500), total IRF3 (catalog # 4302S; 1:500), and NFκβ (catalog # 8242P; 1:500). Tubulin (catalog # ab6160; 1:20000; AbCam, Cambridge, MA) or histone (catalog # NB 100-56347; Novus Biologicals, Littleton, CO) were used as loading controls. IgG conjugated with horse radish peroxidase secondary antibodies used were anti-rabbit (catalog # 7074), anti-mouse, (catalog # 7076), and anti-rat (catalog # 7077S) all Cell Signaling Technologies.

### Cell culture

Partially immortalized human hippocampal neurons (38) and an immortalized human microglia cell line (catalog # T0252; Applied Biological Materials, Richmond, BC, Canada) were used for *in vitro* studies. Cells were maintained in growth media in 6-well plates until 80-85% confluent. Neuron growth media was: N2b medium (customized media from Cytivia, Marlborough, MA) with 0.2 μM beta-estradiol (catalog # E4389; Sigma) and 10 μg/mL fibroblast growth factor basic (catalog # GF003AF; Millipore, Burlington, MA) and 1% heat-inactivated fetal bovine serum (FBS; catalog # MT35016CV; Corning, Corning, NY). Microglia growth media was: PriGroIII (catalog # TM003; Applied Biological Materials, Richmond, BC, Canada) and 10% non-heat inactivated FBS or DMEM (catalog # BW12741F; Lonza, Quakertown, PA) and 10% heat inactivated FBS for cytosolic dsDNA qPCR experiments. At 60-80% confluence, neurons were changed to differentiation media (NSDM, custom media, Cytiva, Global Life Sciences Solutions, Marlborough, MA for 8 d (39)). On differentiation day 9 for neurons and at 80-85% confluence for microglia, media was changed to treatment media (differentiation media without insulin for neurons and growth media without FBS for microglia) 5 h prior to experimental treatments. Following this, cells were treated with either palmitate alone (62.5 μM in microglia or 250 μM in neurons) or palmitate plus insulin (50 nM, both cell types) for 24 h (35, 40). At 24 h, cultures were washed, and cells were fixed for cytosolic dsDNA determination *via* immunocytochemistry or qPCR (below) or isolated for cGAS/STING pathway protein determination by WB (above).

### Cytosolic dsDNA *via* qPCR

Cytosolic DNA isolation was performed as previously published (41). In brief, cells were lysed with RIPA buffer (Invitrogen, Waltham, MA), centrifuged (10 min, 4°C, 700*g*), and supernatant used to quantify and normalize protein concentrations. The pelleted nuclei/whole cell fraction was saved for downstream analysis. Normalized protein concentrations of the supernatant were spun further (30 min, 4°C, 10,000*g*) and the pellet (cytosolic fraction) saved. The pelleted nuclei/whole cell fractions and the pelleted cytosolic fractions were used to isolate DNA using a commercially available kit (catalog # 80004, All prep DNA, RNA, and Protein mini kit; Qiagen, Germantown, MD). Nuclear (18S; 5’-TAG AGG GAC AAG TGG CGT TC-3’ [forward] and 5’-CGC TGA GCC AGT CAG TGT-3’ [reverse]) and mitochondrial DNA (cytochrome oxidase I; 5’- GCC CCC GAT ATG GCG TTT-3’ [forward] and 5’- GTT CAA CCT GTT CCT GCT CC -3’ [reverse]) were run on the nuclei/whole cell fractions and pelleted cytosolic fractions using qPCR SYBR green primers (above). Levels of cytosolic DNA were quantified using the ddC_T_ method (42), with the nuclei fraction used to normalize the cytosolic fraction and the mean ΔC_T_ of the BSA controls as the calibrator for all samples.

### Puzzle box

To assess possible changes in cognition, we performed a modified version of the puzzle box task (43). In this task, mice are intrinsically motivated to move from the light area of the puzzle box into the dark area. On day 2 of diet, puzzle box testing was carried out over a period of 3 d, with a series of three single tasks repeated for a total of three replicates over the first 2 d. The single tasks were then combined into a ‘complex’ task, which was performed once on day 2 and 24 h later on day 3. Latency to ‘escape’ or to enter the dark area of the box was recorded for each of the tasks. Animals were allowed 5 min to perform each task. If the mouse was unable to escape the light area of the box after 5 min, it was removed from the box and its time recorded as 5 min.

### Statistical analysis

We previously established that a sample size of n=8 per group (30, 44) provides adequate power to detect significant metabolic differences between groups. Statistical analyses were performed using Prism 9 (GraphPad Software, La Jolla, CA) using either t-test or one-way analysis of variance (ANOVA) followed by Tukey’s multiple comparisons. Alternatively, analysis of microglial morphology and CNS immunophenotyping data was performed using SAS 9.4 (SAS Institute, Cary, NC) using the Proc Mixed function. Anderson-Darling, D’Agostino-Pearson omnibus, Shapiro-Wilk, and Kolmogorov-Smirnov tests were used to determine normality, and non-normal data was log transformed to achieve normality. Statistical tests and software used for each analysis (glucose tolerance test, immunophenotyping, etc.) and the corresponding results section/figure are detailed in **Supplemental Table 1**. Statistical significance was defined as *p*<0.05 and trends as *p*<0.10. Unless otherwise indicated, results are presented as mean ± standard error of the mean (SEM).

## Results

### Acute HFD impairs metabolic but not cognitive responses

**Figure 1:**
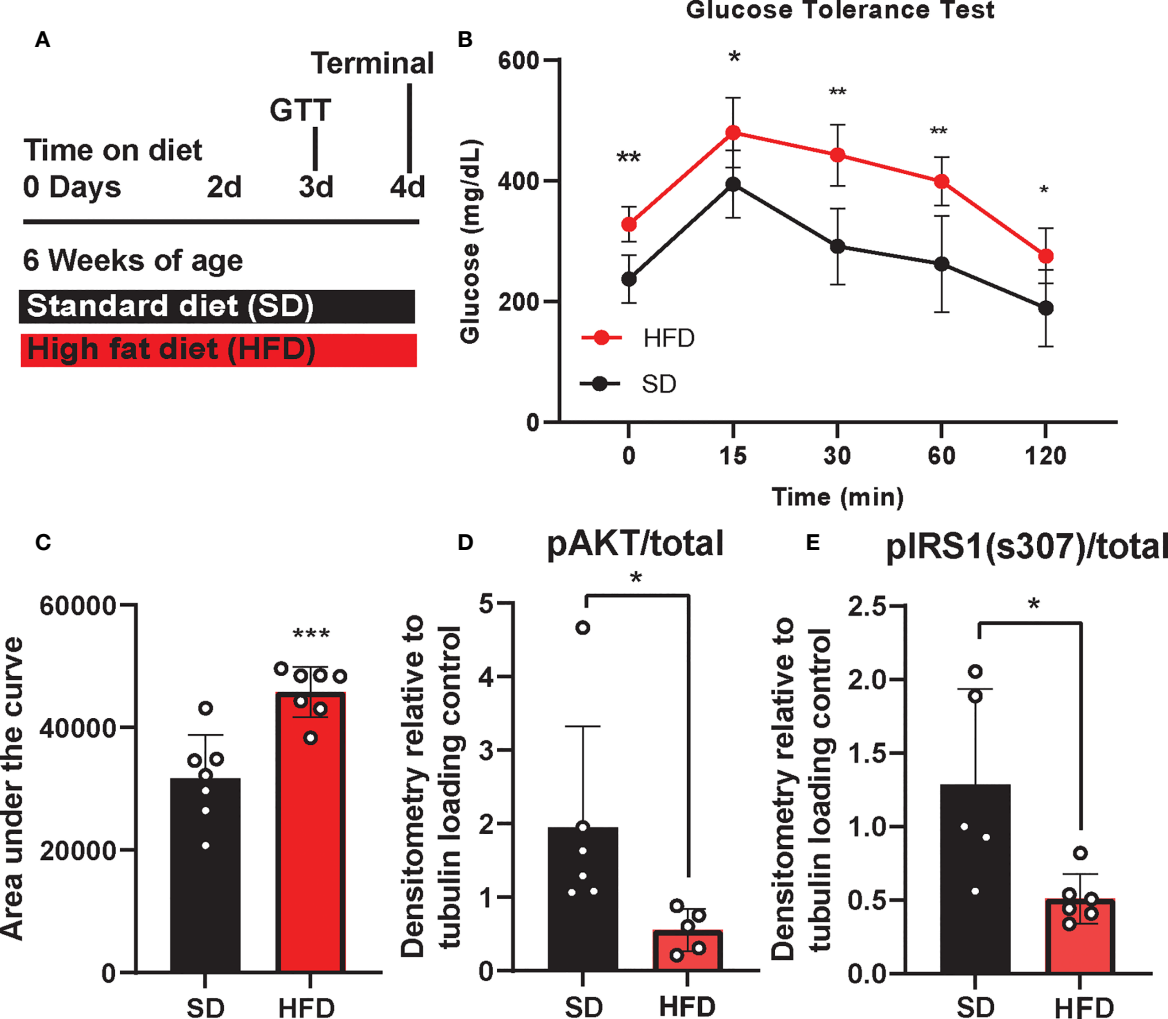
Experimental timeline and metabolic phenotyping. Experimental timeline **(A)**, glucose tolerance test, (GTT; **B**), area under the curve **(C)**, and cortex response to insulin stimulation **(D, E)** in male BL/6 mice fed standard diet (SD) or high fat diet (HFD). Protein expression normalized to tubulin and relative to unstimulated control; *p<0.05, **p<0.01, ***p<0.001.

We previously showed that chronic HFD induces obesity and prediabetes (29), however little is known about the acute metabolic, inflammatory, and cognitive effects of HFD. Therefore, we examined the impact of acute HFD on both metabolic and cognitive function. To do so, BL6 mice were placed on either a HFD or a sucrose matched 10% fat standard diet (SD) for 4 d. GTT was performed on 3 d and mice were harvested for blood and tissue analysis on 4 d (**Figure 1A**). Within just 3 d, we observed HFD impaired glucose tolerance, with higher blood glucose levels at all time points of the glucose tolerance test, as well as a higher area under the curve versus SD mice (**Figures 1B, C** **)**. We and others also previously observed CNS insulin resistance in mice following chronic HFD feeding (29, 45). However, changes in response to acute HFD were unknown. To investigate this, we measured the responsiveness of *ex vivo* brain tissue to insulin by assessing phosphorylation of critical insulin signaling proteins (46, 47). After 3 d of HFD feeding, we observed changes in cortex insulin sensitivity, with decreased phosphorylated protein kinase B (pAkt)/total Akt (**Figure 1D** **;** **Supplemental Figure S1A**) and decreased insulin receptor substrate 1 (IRS1) phosphorylation [pIRS-1(S307)]/total IRS-1 in response to insulin stimulation (**Figure 1E** **;** **Supplemental Figure S1B**).

In addition to metabolic shifts, we and others have shown that chronic HFD also induces cognitive impairment (29, 48, 49), although cognitive changes in response to acute HFD were less clear. Here we performed puzzle box testing, a behavioral task which primarily tests executive function, to assess possible changes in cognition after 3 d on diet. However, we did not detect any differences in behavior between HFD and SD mice (**Supplemental Figure S2**). Overall, 3 d of HFD induces systemic and central metabolic changes related to glucose tolerance and insulin sensitivity, without a detectable impact on cognition within this timeframe.

### Acute HFD alters peripheral and central immune cell populations

We and others have previously reported that chronic HFD also induces changes in circulating inflammatory profiles (33, 48). Using ELISA to examine inflammatory cytokine concentrations and flow cytometry to examine circulating and CNS immune cell populations, we observed changes to plasma inflammatory profiles after 4 d of HFD similar to those seen in long-term HFD feeding (**Supplemental Figure S3**). Specifically, HFD mice had a trending increase in the number of CD4 T-cells (**Supplemental Figure S3C**), and a significant increase in B-cells (number and % of leukocytes; **Supplemental Figures S3F, G**) versus SD animals. HFD mice also had a trend for lower Ly6C+ monocytes compared to SD mice (**Supplemental Figure S3J**). There was no difference due to diet in any of the other measured immune cell populations including CD8 T cells, natural killer cells, Ly6C+ monocytes, Ly6C- monocytes, or neutrophils (**Supplemental Figures S3A, B, D, E, G-I, K-S**). We also measured plasma inflammatory cytokine levels in HFD and SD mice after injection with either saline or lipopolysaccharide (LPS). LPS robustly increased circulating TNF-α and MCP-1 concentrations (**Supplemental Figure S4**); there was no effect of diet.

**Figure 2:**
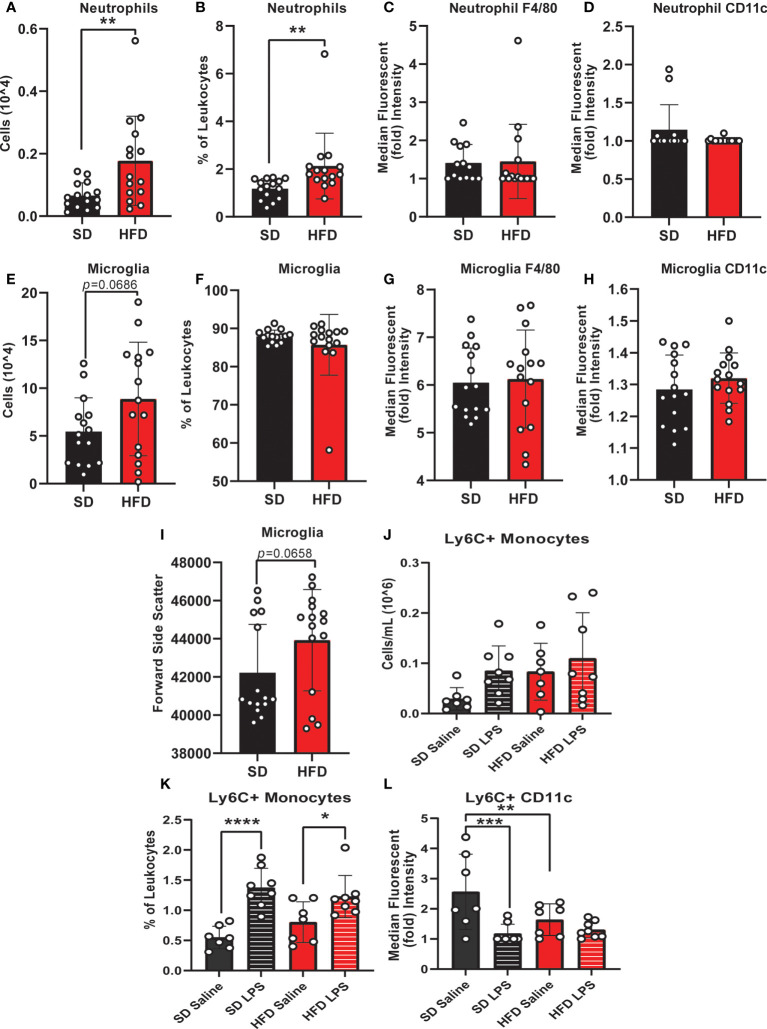
CNS immunophenotyping of myeloid cells by flow cytometry. Data represented as neutrophils (number of cells, % of cells, F4/80 expression, and CD11c expression; **A-D**), microglia (number of cells, % of cells, F4/80 expression, CD11c expression, and forward side scatter; **E-I**), and Ly6C+ monocytes (number of cells, % of cells, and CD11c expression; **J-L**) in male BL/6 mice fed standard diet (SD) or high fat diet (HFD) who were administered saline or LPS (lipopolysaccharide). In the absence of differences between saline and LPS, data for each dietary group were combined and are presented as SD *vs.* HFD alone; *p<0.05, **p<0.01, ***p<0.001, ****p<0.0001.

To understand CNS specific changes in immune cell populations, we repeated our experiment in a separate cohort of HFD versus SD mice, both in control treated (saline injection) and in response to immune challenge (LPS injection). When lymphoid populations in the CNS were examined, HFD increased leukocytes (**Supplemental Figures S5A, B**) and decreased CD8 T-cells (% of leukocytes; **Supplemental Figure S5D**) versus SD animals and LPS injection had no effect (**Supplemental Figure S5**). CD4 T-cell levels were low/not detectable and HFD did not impact the numbers or percentages of CNS natural killer cells (**Supplemental Figures S5C, E-H**). In CNS myeloid cell populations (**Figure 2** and **Supplemental Figure 6**), total immune cell levels and surface marker expression were impacted by 4 d HFD. Neutrophil, microglia, and Ly6C+ monocyte levels were examined as well as expression of CD11c and F4/80, markers of activation and differentiation. HFD mice had more neutrophils (numbers and %; **Figures 2A, B** **)** and a greater number of microglia, which also had a trending increase in size as measured by a larger forward side scatter (**Figures 2C, I** **)** suggesting activation. There were no differences due to LPS treatment or due to diet for neutrophil or microglial F4/80 or CD11c expression, or for the percentage of microglia (**Figures 2C, D, F-H**). LPS administration did impact LyC6+ monocyte numbers and surface expression of CD11c, which was further altered by diet (**Figures 2K, L** **)**.

Monocyte CD11c expression can indicate a change in monocyte activation, and activation can promote monocyte differentiation into a microglial-like phenotype (32, 50, 51). We observed that LPS increased CNS monocytes in both HFD and SD animals; however, monocyte CD11c expression was lower in response to saline injection in HFD versus SD mice. In contrast, LPS decreased monocyte CD11c expression in SD but not HFD mice. Increased numbers of microglia and decreased expression of CD11c on monocytes in the absence of increased monocyte numbers likely indicates that HFD promotes monocyte conversion into a more microglial-like phenotype, which LPS fails to further promote. Cumulatively, our findings indicate that acute HFD of only 3 d produces changes in peripheral and central immune cell populations. In this setting, LPS stimulation differentially impacts CNS immune cell dynamics, *i.e*., monocyte to microglial shifts, in HFD versus SD.

### Acute HFD activates hippocampal microglia

**Figure 3:**
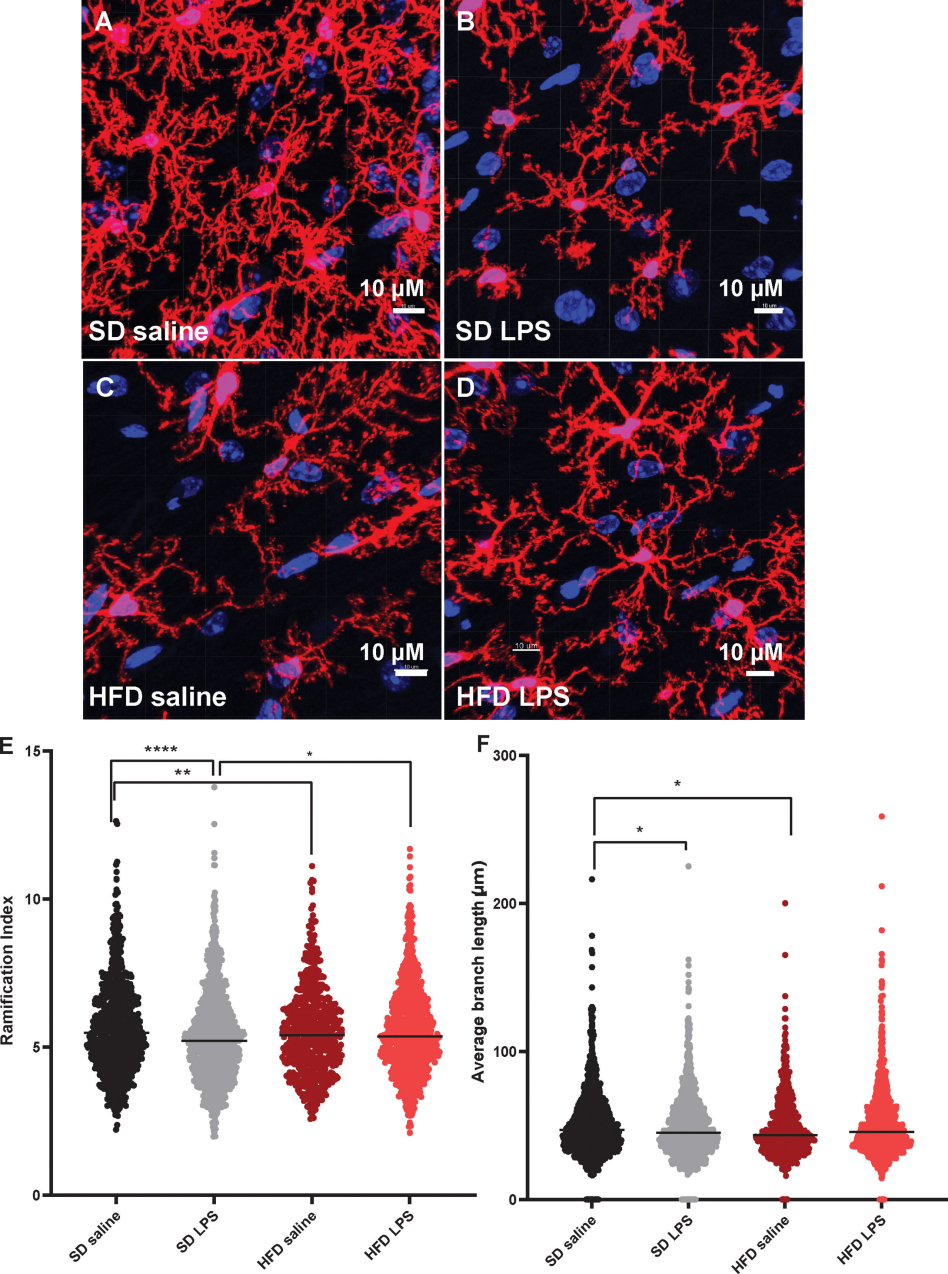
Microglial morphology. Representative images of IBA-1 microglia (red stain) in male BL/6 mice fed standard diet (SD) or high fat diet (HFD) who were administered saline or LPS (lipopolysaccharide; **A-D**). Quantification of microglia ramification index **(E)** and average branch length **(F)**. In the absence of differences between saline and LPS, data for each dietary group were combined and are presented as SD *vs.* HFD alone; *p<0.05, **p<0.01, ****p<0.0001.

Since we observed changes in microglia numbers and size upon 4 d of HFD in our CNS immunophenotyping data, we were interested in further interrogating acute inflammatory microglial changes. We therefore assessed microglial morphology (34) as a proxy of activation in an area of the brain critically important for learning and memory, the hippocampus. Mice were administered HFD or SD with or without LPS stimulation for 4 d, and microglia morphology was examined using confocal microscopy. Three days of HFD shifted the morphology of hippocampal microglia to a state indicative of activation (**Figures 3A–D**) where microglia of HFD mice given saline (**Figure 3C**) appeared to have a larger soma size and more ameboid-like shape with fewer and shorter processes compared to microglia of SD mice given saline (**Figure 3A**). Additionally, microglia in SD (**Figure 3B**) and HFD **(** **Figure 3D**) mice given LPS appeared to take on an activated morphology similar to HFD mice given saline. Indeed, when quantifying these morphological changes, we observed that HFD lowered the ratio of three-dimensional space occupied by the microglia to its perimeter (ramification index; **Figure 3E**) versus SD mice. Interestingly, administering LPS to SD mice caused the microglia to have a decreased ramification, indicating activation. However, administering LPS to HFD mice did not change their ramification index; this inability of HFD microglia to respond to LPS stimulation may suggest they are activated under basal conditions to such a degree that further stimulation cannot provoke an appropriate immunological response to cellular insult or injury. Like the ramification index, HFD microglia had shorter average branch length (**Figure 3F**), shorter maximum branch length (**Supplemental Figure S7D**), and shorter minimum branch length versus SD microglia (**Supplemental Figure S7F**). LPS stimulation did not affect territorial volume (**Supplemental Figure S5A**), average branch length (**Figure 3F**), and maximum branch length (**Supplemental Figure S7D**). While HFD mice had a greater overall cell volume compared to SD mice, there no effect of LPS (**Supplemental Figure S7B**). Between groups differences in the number of microglial branch points and end points were varied and dependent upon hippocampal region (**Supplemental Figures S7C, E**). Thus, acute 3 d HFD activates hippocampal microglia and renders them less able to mount a response to additional stimulation, *e.g*., to LPS.

### Acute HFD activates cGAS/STING signaling

**Figure 4:**
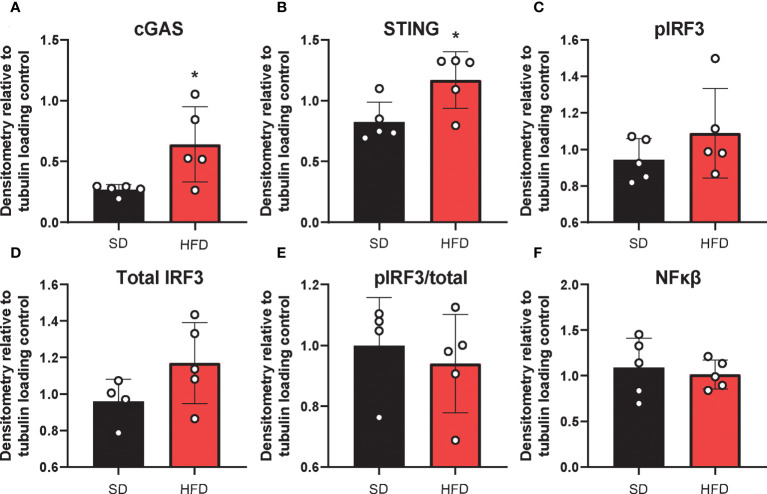
Hippocampal cGAS/STING protein expression. Expression in male BL/6 mice fed standard diet (SD) or high fat diet (HFD). Data represented as cGAS **(A)**, STING **(B)**, pIRF3 **(C)**, total IRF3 **(D)**, pIRF3/total **(E)**, and NFκβ **(F)** relative protein expression. Protein expression quantified as average band intensity relative to tubulin loading control; *p<0.05.

The deleterious role of cGAS/STING inflammatory signaling in obesity and metabolic dysfunction is well established in the periphery, particularly in adipose tissue) (19, 21). However, little is known about its role in this context in the CNS. Therefore, next we wanted to establish the effects of HFD feeding on hippocampal cGAS/STING pathway protein expression (**Figure 4** **;** **Supplemental Figure S8**). To do so, we took hippocampal tissue from SD and HFD animals fed diet for 4 d, homogenized it, and performed Western Blotting. We observed that HFD of only 4 d already acutely upregulated expression in the hippocampus of the dsDNA sensing cGAS and its adaptor molecule STING (**Figures 4A, B** **;** **Supplemental Figures S8A, B**). However, HFD did not promote phosphorylation or change expression of the cGAS/STING pathway transcription factors IRF3 (**Figures 4C–E** **;** **Supplemental Figures S8C–E**) and NFkβ (**Figures 4F** **;** **Supplemental Figures S8C–E**). When activated, IRF3 and NFkβ act as canonical transcription factors and move from the cytosol to the nucleus to induce gene transcription. Therefore, a lack of changes these transcription factors in bulk tissue is perhaps not surprising. Differences in cytosolic *vs.* nuclear localization are likely present, as have been observed by others in culture and in microglia (21, 26). Together, these data further suggest an early upregulated and pro-inflammatory phenotype involving the cGAS/STING pathway after only 3 d on HFD diet.

**Figure 5:**
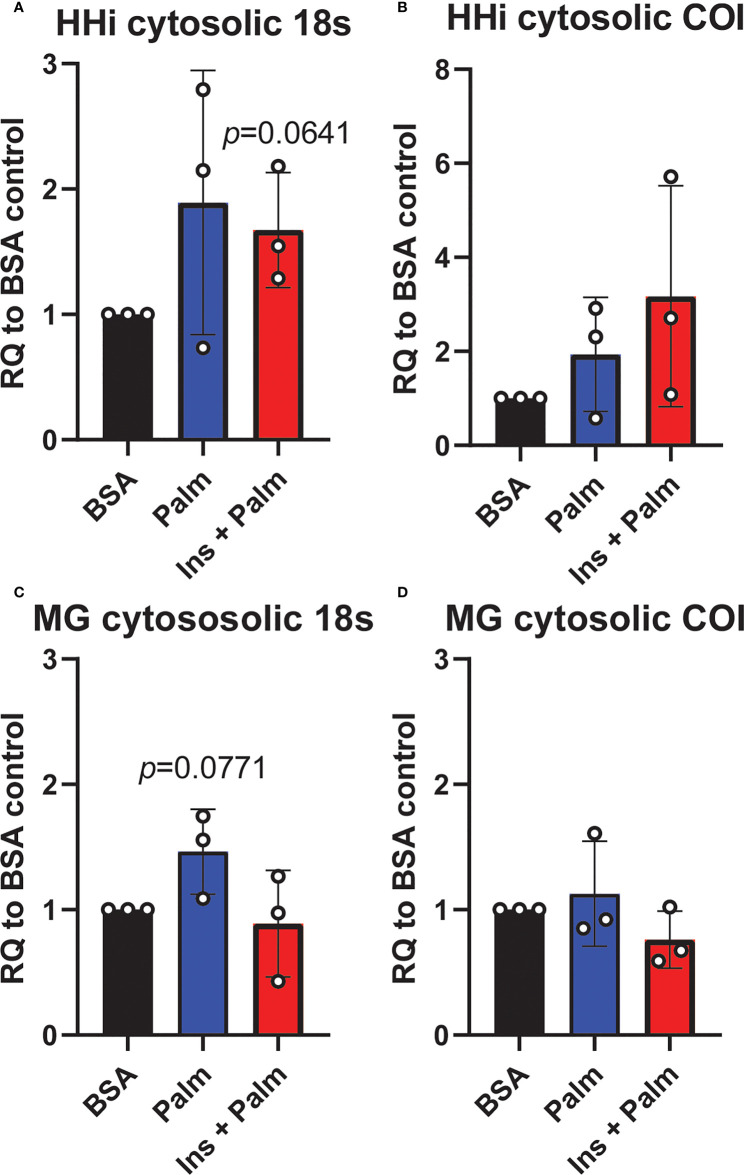
Cytosolic DNA concentrations. Relative quantity (RQ) of cytosolic DNA (nuclear and mitochondrial) in partially immortalized human hippocampal neurons (HHi; n=3, **A, B**) and in a human microglial cell line (MG; n=3, **C, D**). Cells treated with palmitate (Palm; HHi=250 μM, microglia=62.5 μM, 24h) or a combination of insulin and palmitate (Ins + Palm; above palmitate concentrations + 50 nM insulin, 24h). Values relative to BSA controls.

**Figure 6:**
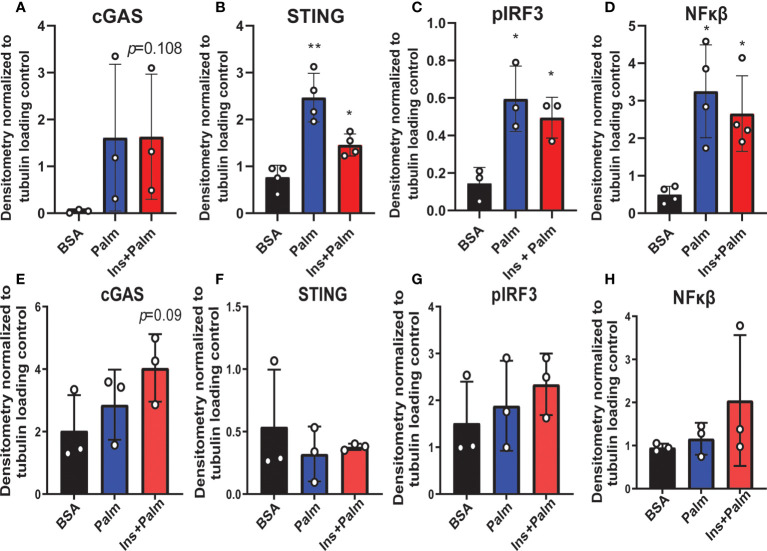
Neuronal and microglial cGAS/STING protein expression. Expression in a human microglial cell line (n=3 biological replicates, **A-D**) and a human hippocampal neuronal cell line (n=3 biological replicates, **E-H**) treated with either palmitate alone (Palm; HHi=250 μM, MG=62.5 μM, 24h) or a combination of insulin and palmitate (Ins+Palm; above concentrations of Palm+50nM insulin, 24h). Relative protein expression quantified as average band intensity relative to tubulin loading control; *p<0.05, **p<0.01.

We previously showed that *in vitro* treatment of neurons with insulin or palmitate for 24 h produces insulin resistance, providing a cell culture model of prediabetes, with the expected changes in cellular signaling pathways (40, 52). We adopted this same approach to establish the contribution of various CNS cell types, namely neurons and microglia, to cGAS/STING pathway activation. Using a partly immortalized human hippocampal cell line and an immortalized human microglial cell line, we first established the presence of cytosolic DNA in response to palmitate and insulin treatment. Our data show a trending increase in cytosolic nuclear DNA (18s) in both neurons and microglia in response to palmitate or combined insulin and palmitate treatment (**Figures 5A, C** **)**. However, there were no differences in either cell type in response to stimulation for cytochrome oxidase I DNA, a marker of mitochondrial DNA (**Figures 5B, D** **)**. Of note, only trending differences in cytosolic nuclear DNA and a lack of differences in mitochondrial DNA were likely due to low sample sizes and a high degree of variability between replicates. Future studies could address how obesogenic conditions might cause genomic damage and the role of mitochondrial *vs.* genomic or nuclear damage on cGAS/STING signaling in the CNS. We next assessed cGAS/STING pathway protein expression in both cell types. There was a robust response in microglia, with a significant increase in STING, pIRF3, and NFκβ, in the presence of either palmitate alone or combined insulin and palmitate for 24 h (**Figure 6** **;** **Supplemental Figure S9**). We also found a trending increase in cGAS protein expression in response to acute treatment for 24 h with either palmitate alone or combined insulin and palmitate in hippocampal neurons (**Figure 6A** **;** **Supplemental Figure S9A**).

**Figure 7:**
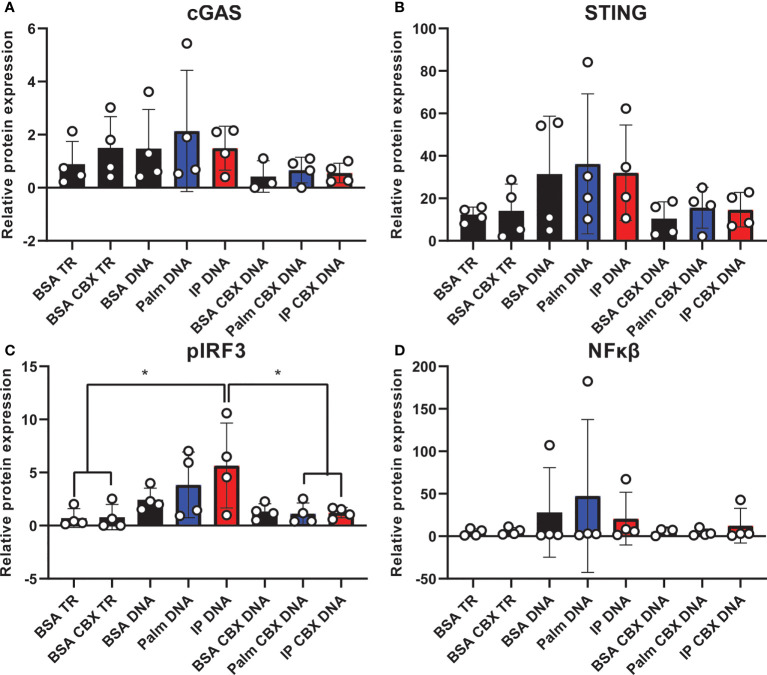
Co-culture cGAS/STING protein expression with and without gap junction inhibitor. Expression in a human hippocampal and human microglial cell line co-culture (n=4). Cells pretreated with bovine serum albumin (BSA; 31.25 µM, 24h) as a control, palmitate (Palm; 31.25 µM, 24h), or a combination of insulin and palmitate (IP; 31.25 µM palmitate and 50 nM insulin, 24h) +/- the gap junction inhibitor carbenoxolone (CBX; 150 µM), then stimulated with the dsDNA analog Poly dA:dT (DNA; 1µg/mL). Protein expression of cGAS **(A)**, STING **(B)**, pIRF3 **(C)**, and NFκβ **(D)** quantified quantified as average band intensity relative to histone loading control; *p<0.05. TR, transfection reagent.

Finally, we assessed cGAS/STING pathway activation in co-culture to evaluate the contribution of inflammatory crosstalk on potential pathological mechanisms *via* gap junctions in the CNS. Inflammatory crosstalk (53) is vital for normal intercellular communication (54). However, aberrant inflammatory crosstalk in the CNS (either *via* glia-glia or glia-neuron signaling) may promote pathological inflammatory mechanisms. Indeed, it plays a role in neurodegenerative diseases, such as AD/ADRD (55–57), and gap junctions facilitate transfer between cells of the cGAS/STING second messenger, cyclic GMP-AMP (cGAMP) (23, 56). To determine whether gap junctions mediate inflammatory crosstalk, we co-cultured neurons and microglia in the presence or absence of a gap junction inhibitor (CBX; carbenoxolone). Co-cultures were pre-treated with either the saturated fatty acid palmitate or the combination of insulin to mimic obesogenic prediabetic conditions. Our data (**Figure 7** **;** **Supplemental Figure S10**) show that treating co-cultures with transfection reagent alone did not change cGAS (**Figure 7A** **;** **Supplemental Figure S10A**), STING (**Figure 7B** **;** **supplemental Figure S10B**), or NFκβ protein expression (**Figure 7D** **;** **Supplemental Figure S10D**) in the presence of the gap junction inhibitor, carbenoxolone. However, we observed a significant increase in co-cultures pre-treated with insulin and palmitate for 24 h then stimulated with the dsDNA analog, poly dA:dT (poly deoxyadenylic-deoxythymidylic acid sodium salt), which was completely reversed in the presence of carbenoxolone (**Figure 7C** **;** **Supplemental Figure S10C**). In aggregate, these data suggest that cGAS/STING inflammatory crosstalk between CNS cells, *e.g*., neurons and microglia, in response to metabolic injury is mediated, at least in part, by gap junctions.

## Discussion

Metabolic dysfunction, in the form of chronic obesity, prediabetes, or diabetes, induces peripheral and central inflammation which correlate with cognitive impairment (58, 59). However, early inflammatory events secondary to obesity- or prediabetes that might contribute to cognitive impairment remain uncertain. The innate immune cGAS/STING pathway is dysregulated in cognitive impairment and neurogenerative disease (24, 26) and by responding to excess saturated fatty acids may connect metabolic dysfunction to inflammation in the CNS (19–21). In the current study, we examined the effect of acute HFD on peripheral and CNS inflammation, cognition, and CNS cGAS/STING activation. Our data show that acute HFD for only 3 d causes peripheral and central metabolic and immunologic changes indicative of insulin resistance and an acute pro-inflammatory response, though changes in cognition were not detected. Additionally, acute HFD activates CNS microglia, as measured by changes in cell size and morphology, and promotes cGAS/STING signaling. This immune response was mirrored *in vitro* under conditions of metabolic injury, particularly in microglia, as well as in in neuron-microglia co-culture and was blocked by a gap junction inhibitor. Overall, our findings indicate that inflammation and cGAS/STING activation are early responses to HFD, potentially through direct gap junction-mediated neuron-microglia crosstalk in the CNS.

We found that short-duration HFD induced acute peripheral and CNS metabolic changes in mice, specifically impaired glucose tolerance and insulin resistance. These findings are aligned with another study of 3 d of HFD feeding, which similarly saw impaired glucose homeostasis (60). These changes are also consistent with literature regarding chronic HFD, *i.e*., of a few to several weeks, that report increases in body weight and impaired glucose tolerance (29, 30). We further show that both peripheral and CNS immune cell populations are dysregulated after only 3 d on HFD. Specifically, HFD increased circulating and CNS lymphocytes and neutrophils. We also observed that acute HFD decreased circulating Ly6C+ monocytes and Ly6C+ monocytes in the CNS had lower CD11c expression. Concurrent with increased CNS microglia, these data suggest that HFD promoted of monocyte recruitment to the CNS and monocyte conversion to a more microglia-like phenotype. Moreover, LPS failed to mount a further immune response in HFD, indicating peripheral and CNS immune cells are activated to such a degree by HFD that LPS is unable to provoke an appropriate response. Our findings are broadly aligned with the acute impact of HFD on the CNS, where others have reported increased levels of inflammatory cytokines after 3 d on diet (12). Furthermore, it is frequently reported that chronic HFD feeding induces an inflammatory phenotype (33, 48, 61).

HFD-induced pro-inflammatory responses through upregulated cGAS/STING signaling in peripheral tissues has been proposed as a potential pathological mechanism in obesity and prediabetes/diabetes (19, 21, 41). As an intracellular pattern recognition receptor, cGAS/STING is widely expressed by innate cells of the CNS, including microglia (62, 63), which canonically senses cytosolic dsDNA of viral or bacterial origin (64). However, the cGAS/STING pathway can also be activated by cytosolic self dsDNA released under conditions of metabolic stress, such as by saturated fatty acid overload (20, 64). Indeed, HFD fed mice have elevated adipose (41) and liver (65) STING levels. In endothelial cultures, the long-chain saturated fatty acid palmitate activates cGAS/STING and induces inflammation (21, 41). Further, STING deficiency partially reverses HFD-induced weight gain, decreases plasma free fatty acids and adipose macrophage infiltration, and improves impaired insulin sensitivity and glucose tolerance (41).

While there is ample evidence to suggest a role for cGAS/STING in obesity and prediabetes/diabetes in the periphery, the role of cGAS/STING in the brain is less clear. We observed cGAS/STING was upregulated in the hippocampus of HFD animals versus SD controls. We previously established that our HFD feeding paradigm induces obesity, prediabetes and cognitive impairment with chronic HFD in mice (29). While here we did not observe cognitive impairment after only 3 d of HFD, our findings suggest HFD promotes an acute and early CNS pro-inflammatory programming that precedes or initiates the cascade of processes leading up to neurodegeneration and cognitive impairment with chronic HFD. Conversely, others have reported changes in cognition after acute HFD feeding (66–68). Differences may have arisen from variations in model system (mouse versus rat), animal age (5 wk versus 12 wk) or testing modality (puzzle box versus contextual fear conditioning versus radial arm maze) (66–68). Moreover, it is possible that cognitive differences in HFD versus SD animals in only measurable upon additional stimulation, *e.g*., by LPS (66). Therefore, the temporal evolution of cognitive impairment upon acute HFD requires further study.

In alignment with our findings of early cGAS/STING activation, cGAS/STING is implicated in frank dementia, such as AD/ADRD (24, 26). In the brains of AD models, cGAS/STING is increased and improving DNA damage/repair by NAD+ supplementation normalizes cGAS/STING levels, reduces inflammation, and improves behavioral outcomes (24). Furthermore, cGAS/STING may be involved in AD *via* interaction with one of the key pathological AD proteins, tau. Specifically, tau activates cGAS/STING *via* binding to polyglutamine binding protein 1, which is essential for tau-mediated cGAS/STING activation, specifically in microglia (26). In a Parkinson’s disease mouse model, knocking out cGAS/STING signaling rescues the inflammatory phenotype, prevents loss of dopaminergic neurons, and improves motor deficits (27). In amyotrophic lateral sclerosis, the critical disease protein TDP-43 promotes the release of mitochondrial dsDNA into the cytosol, which subsequently activates the cGAS/STING pathway and promotes neurodegeneration (28).

We observed that acute HFD was sufficient to activate hippocampal microglia, which were unable to respond to additional stimulus in the form of LPS injection. Further, using an established *in vitro* model of metabolic injury, we observed a stronger response of the cGAS/STING pathway in microglia compared to neurons. This was anticipated, as cGAS/STING pathway proteins are highly expressed in microglia (62). Moreover, as we observed, HFD induces an inflammatory phenotype in hippocampal microglia (11, 12), and inflammatory microglia play critical roles in AD/ADRD pathology and related neuroinflammation (69–71). cGAS/STING activation primarily results in type 1 interferons (IFN) pro-inflammatory cytokine production, which acts to further stimulate cytokine release, *e.g*., of IL-1β, IL-6, TNF-α (72). Excessive cGAS/STING activation contributes to pathological mechanisms, often mediated in the CNS by microglia (72, 73). This cGAS/STING activation and subsequent IFN release structurally and functionally injures neurons (72). Our findings indicate that microglia may be constituently activated under HFD conditions in the hippocampus, are less able to respond to inflammatory stimulus, and may contribute to CNS neuroinflammation, neurodegeneration, and eventual cognitive decline.

The immune system has multiple functions, including to induce inflammation, recruit immune cells, initiate protective cellular programs (including metabolic processes), preserve homeostasis, and maintain tissue functions (74). To perform these functions, it partly relies on inflammatory crosstalk, such as gap junctions (53), for intercellular communication (54). This crosstalk may become dysregulated upon chronic inflammatory activation, such as occurs in obesity and prediabetes, and thus is a potential mechanism promoting disease progression. In our co-culture model of human hippocampal neurons and microglia, we showed activation of the cGAS/STING pathway is strongly reduced in the presence of a gap junction inhibitor. These data show that gap junction mediated cGAS/STING crosstalk is a mechanism by which cGAS/STING inflammatory signaling can be promoted in the CNS in the presence of metabolic insults. In fact, gap junctions are relevant to neurodegenerative diseases, such as AD. Gap junctions are elevated near Aβ plaques (75, 76), and their blockade slows disease progression (55). Further, immune responses and cytokines can regulate gap junctions during insult, infection, or injury (77, 78). cGAS/STING has been shown to utilize gap junctions as an inflammatory crosstalk mechanisms in HEK cells and murine fibroblasts (23). Specifically, in response to cytosolic dsDNA, cGAS triggers production of its second messenger, cGAMP (20), which can travel to neighboring cells *via* gap junctions and stimulate downstream cytokine production by activating STING and pIRF3 (23). This represents a source of direct cell-to-cell crosstalk, contributing to inflammatory activation in neighboring cells, possibly furthering pathological processes. While our data support a role for gap junctions in promoting inflammatory crosstalk, it is unclear which cell types are the primary source of this inflammation. Future studies using single cell sequencing and cGAS cell specific knock out models are currently underway to better understand how different cell types contribute to this inflammation and the downstream effects they might have on cognition.

However, our study had some limitations. First it was carried out in male animals only. We (79) and others (80) have shown that male and female animals have sexually dimorphic responses to high fat diet feeding, particularly early in the paradigm. Additionally, there are known differences between males and females in terms of immune function and inflammation (81, 82), including in microglia (83). These differential effects also potentially impact cognition, as some have shown a differential effect of sex on cognitive outcomes (84). As mentioned above, no differences were observed between groups for puzzle box performance. However, motivation to escape in the puzzle box task is primarily driven by the animal’s fear and anxiety in brightly lit spaces (85). Additional non-cognitive tasks that more directly measure anxiety under a similar motivation, such as the open field task (86), would allow for discrimination between a lack of cognitive deficits *vs.* overall anxiety in the animals and should be considered for future studies.

Overall, our data indicate that acute HFD feeding promotes early dysregulated glucose and insulin metabolism in the periphery and CNS. HFD feeding also causes an acute pro-inflammatory response, including microglial and innate inflammatory cGAS/STING pathway activation in the brain. Our *in vitro* data in neurons and microglia further point to a critical role for microglia in promoting this pro-inflammatory phenotype and indicate that gap junction may, at least in part, mediate cGAS/STING signaling, participating in inflammatory spread in the CNS.

## Data availability statement

The original contributions presented in the study are included in the article/**Supplementary Material**. Further inquiries can be directed to the corresponding author.

## Ethics statement

The animal study was reviewed and approved by The University of Michigan’s Institutional Animal Care and Use Committee approved all animal protocols (PRO0010039).

## Author contributions

SE, RH, and EF designed the studies. IW-D, ST, and BM performed the immunophenotyping. BK, JH, FM, IW-D, ST, RH, and SE contributed to the tissue processing. BK and SE performed the *ex vivo* insulin stimulation and western blotting. IW-D sectioned and stained the images for microglial morphology. SE and RH imaged and analyzed the microglial morphology data. CP and SE performed the cell culture and subsequent PCR and western blotting. SE performed statistical analyses. SE wrote the manuscript. RH, EF, BK, and BM edited the manuscript. All authors contributed to the article and approved the submitted version.

## Funding

Funding was provided by the NIH (U01AG057562, U24DK115255, R01DK130913, T32DK007245), the Sinai Medical Staff Foundation Research Fund for Studying Diet and Brain Health, the Robert and Katherine Jacobs Environmental Health Initiative, the Robert E. Nederlander Sr. Program for Alzheimer’s Research, the Andrea and Lawrence A. Wolfe Brain Health Initiative Fund, the A. Alfred Taubman Medical Research Institute, and the NeuroNetwork for Emerging Therapies. SE is supported by an Edith Briskin/SKS Foundation NeuroNetwork Emerging Scholar Fund, the Michigan Alzheimer’s Disease Research Center early career investigator mentorship program (supported by the NIH/NIA funded by the Michigan Alzheimer’s Disease Research Center (P30AG072931) and the University of Michigan Alzheimer’s Disease Center), and NIA K99/R00 (1K99AG071667-01A1).

## Acknowledgments

Authors are grateful to the University of Michigan flow cytometry core and Rogel Cancer Center immunology core for assistance with flow cytometry and ELISA, as well as the University of Michigan Unit for Laboratory Animal Medicine for their care of the animals used for this work.

## Conflict of interest

The authors declare that the research was conducted in the absence of any commercial or financial relationships that could be construed as a potential conflict of interest.

## Publisher’s note

All claims expressed in this article are solely those of the authors and do not necessarily represent those of their affiliated organizations, or those of the publisher, the editors and the reviewers. Any product that may be evaluated in this article, or claim that may be made by its manufacturer, is not guaranteed or endorsed by the publisher.

## Supplementary material

Supplementary Figure 1 Representative insulin signaling western blot images. Representative unaltered images of western blots quantified in main figure 1 (w/link color) for cortex insulin signaling protein expression of AKT (pAKT and total AKT; **A**) and IRS-1 (pIRS-1(pS307) and IRS-1; **B**) in male BL/6 mice fed standard diet (SD) or high fat diet (HFD) and +/- acute insulin treatment.

Supplementary Figure 2 Cognition as measured by puzzle box testing. Data represented as single tasks **(A-D)**, and the combination of the single tasks into a complex task **(E)** in male BL6 mice fed high-fat diet (HFD) versus standard diet (SD).

Supplementary Figure 3 Peripheral immunophenotyping by flow cytometry. Data represented as leukocytes (number of cells **A**), CD4 T-cells (% of cells or number of cells; **B, C**), CD8 T-cells (% of cells or number of cells; **D, E**), B-cells (% of cells or number of cells; **F, G**), natural killer cells (% of cells or number of cells; **H, I**), Ly6C+ monocytes (% of cells or number of cells; **J, K**), Ly6C- monocytes (% of cells or number of cells; **L, M**), neutrophils (% of cells or number of cells; **N, O**), Ly6C+ monocytes CD11c expression (median fluorescent (fold) intensity; **P**), Ly6C+ monocytes F4/80 expression (median fluorescent (fold) intensity; **Q**), Ly6C- monocytes CD11c expression (median fluorescent (fold) intensity; **R**), and Ly6C- monocytes F4/80 expression (median fluorescent (fold) intensity; **S**) in male BL6 mice fed high-fat diet (HFD) versus standard diet (SD); **p*<0.05.

Supplementary Figure 4 Plasma inflammatory cytokines as measured by ELISA. Data represented as plasma TNF-α (pg/mL; **A**) and plasma MCP-1 (pg/mL; **B**) concentrations in male BL6 mice fed high-fat diet (HFD) versus standard diet (SD), administered either saline or lipopolysaccharide (LPS); ***p*<0.01, *****p*<0.0001.

Supplementary Figure 5 CNS immunophenotyping of lymphoid cells by flow cytometry. Data represented as leukocytes (number of cells or % of cells; **A, B**), CD8 T-cells (number of cells or % of cells; **C, D**), CD4 T-cells (number of cells or % of cells; **E, F**), and natural killer cells (number of cells or % of cells; **G, H**) in male BL6 mice fed high-fat diet (HFD) versus standard diet (SD). There were no differences between animals administered saline *vs.* lipopolysaccharide (LPS), therefore SD and LPS animals were combined within their appropriate dietary groups; **p*<0.05.

Supplementary Figure 6 Representative flow cytometry panels for CNS myeloid cells. Data represented as CD11c or F4/80 surface expression on neutrophils **(A)**, microglia **(B)**, and monocytes **(C)** in male BL6 mice fed high-fat diet (HFD; black) versus standard diet (SD; red) given saline. For monocytes, solid lines represent mice given saline and dashed lines represent mice given lipopolysaccharide. Grey peaks represent IgG control antibody.

Supplementary Figure 7 Microglial morphology. Quantification of microglial territorial volume (μm^3^; **A**), cell volume (μm^3^; **B**), number of end points **(C)**, maximum branch length (μm; **D**), number of end points **(E)**, and minimum branch length (μm; **F**) in male BL6 mice fed high-fat diet (HFD) versus standard diet (SD) administered saline or LPS (lipopolysaccharide; **A-D**). Quantification of microglia percentage occupied volume **(E)** and average branch length **(F)**. For A, B, D, and F quantification was performed on individual cells per image (n=3 images for the CA1 and molecular layers of the hippocampus and n=2 images for the hilus). For C and E quantification was performed by combing all images per hippocampal region. In the absence of differences between saline and LPS, data for each dietary group were combined and are presented as SD *vs.* HFD alone; **p*<0.05, ***p*<0.01, *** *p*<0.001, *****p*<0.0001.

Supplementary Figure 8 Representative hippocampal cGAS/STING western blot images. Representative unaltered images of western blots quantified in main figure 4 (w/link color) for hippocampal cGAS/STING pathway protein expression of cGAS **(A)**, STING **(B)**, pIRF3 **(C)**, IRF3 **(D)**, NFκβ **(E)**, and tubulin **(F)** in male BL/6 mice fed standard diet (SD) or high fat diet (HFD).

Supplementary Figure 9 Representative neuron and microglia cGAS/STING western blot images. Representative unaltered images of western blots quantified in main figure 6 (w/link color) for cGAS/STING pathway protein expression of cGAS **(A)**, STING **(B)**, pIRF3 **(C)**, NFκβ **(D)**, and tubulin **(F)** in palmitate (Palm) and insulin (Ins) stimulated neuronal and microglial cell lines. Conditions in bold are those used for analysis.

Supplementary Figure 10 Representative co-culture cGAS/STING western blot images. Representative unaltered images of western blots quantified in main figure 7 (w/link color) for cGAS/STING pathway protein expression of cGAS **(A)**, STING **(B)**, pIRF3 **(C)**, NFκβ **(D)**, and histone **(F)** in in palmitate (Palm) and insulin and palmitate (IP) stimulated neuronal and microglial cell line co-culture +/- the gap junction inhibitor carbenoxolone (CBX; 150 μM). Co-cultures were further stimulated with the dsDNA analog Poly dA:dT (DNA; 1μg/mL). Conditions in bold are those used for analysis. TR; transfection reagent.
