# Supplementary figures and images for: cGAS/STING and innate brain inflammation following acute high-fat feeding
Source: Front Immunol. 2022 Sep 29;13:1012594. doi: 10.3389/fimmu.2022.1012594 (PMC9556783; doi:10.3389/fimmu.2022.1012594)

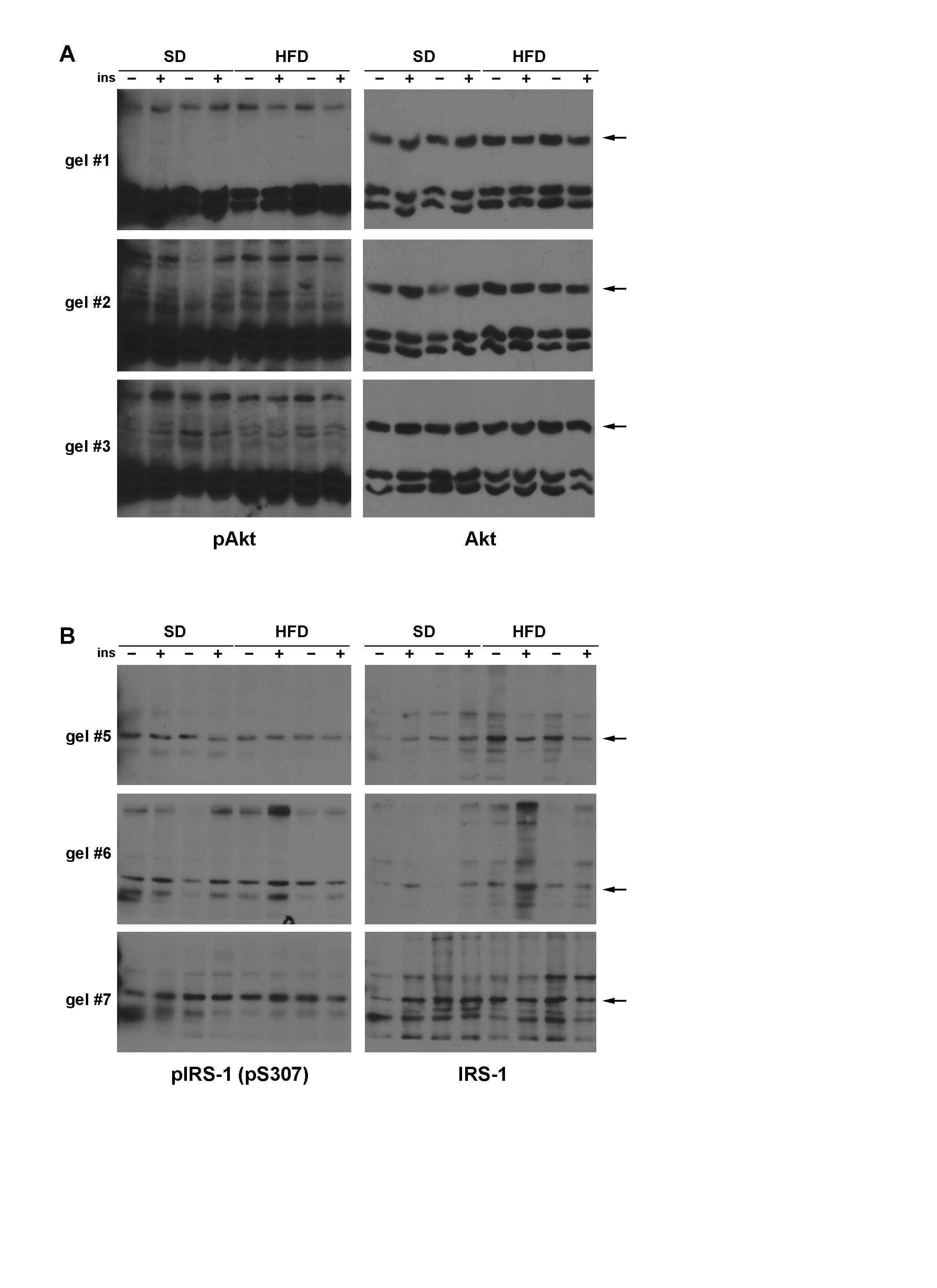

Supplement: Supplementary Figure 1 — Representative insulin signaling western blot images. Representative unaltered images of western blots quantified in main figure 1 (w/link color) for cortex insulin signaling protein expression of AKT (pAKT and total AKT; A) and IRS-1 (pIRS-1(pS307) and IRS-1; B) in male BL/6 mice fed standard diet (SD) or high fat diet (HFD) and +/- acute insulin treatment. [file Image_1.tif]

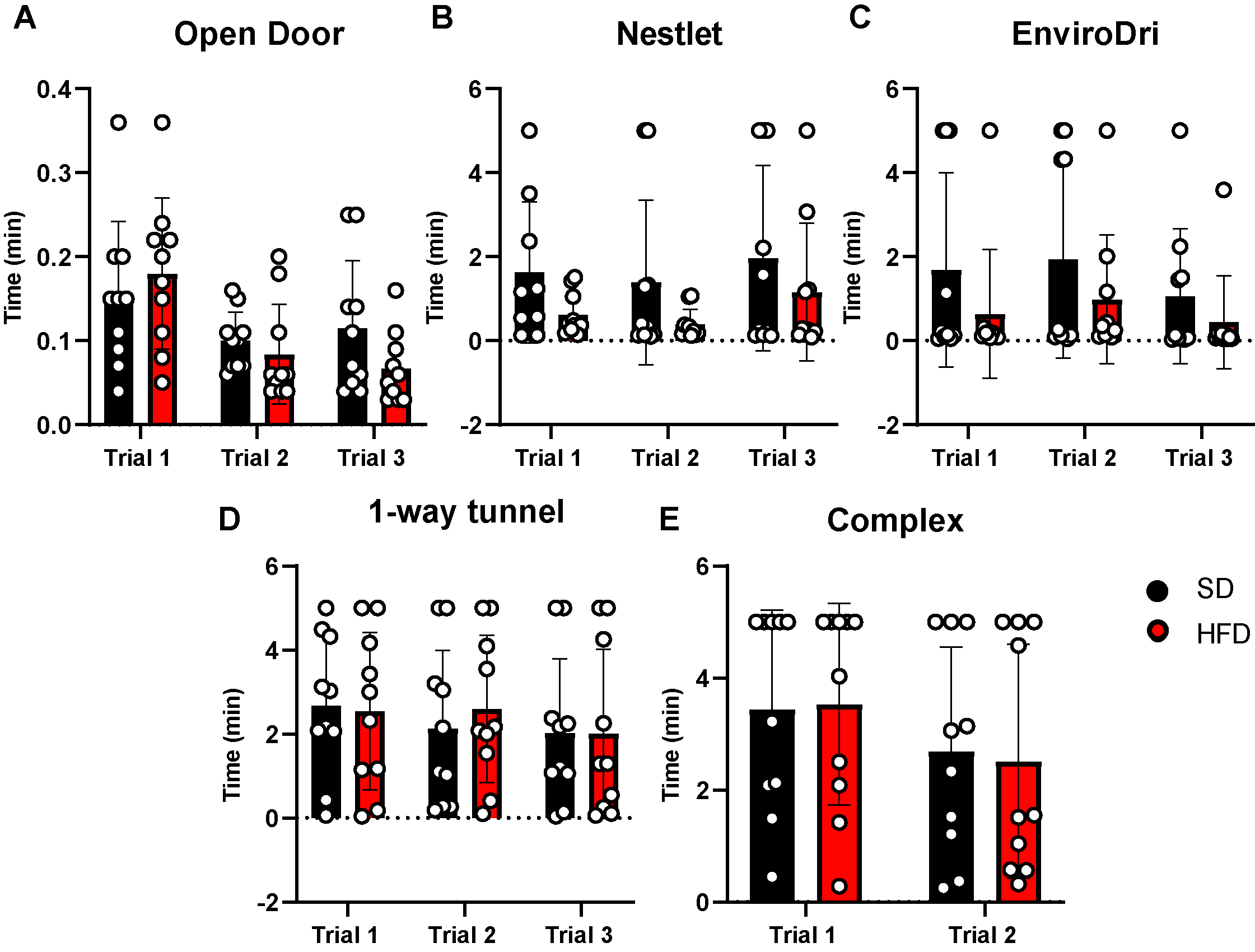

Supplement: Supplementary Figure 2 — Cognition as measured by puzzle box testing. Data represented as single tasks (A-D), and the combination of the single tasks into a complex task (E) in male BL6 mice fed high-fat diet (HFD) versus standard diet (SD). [file Image_2.tif]

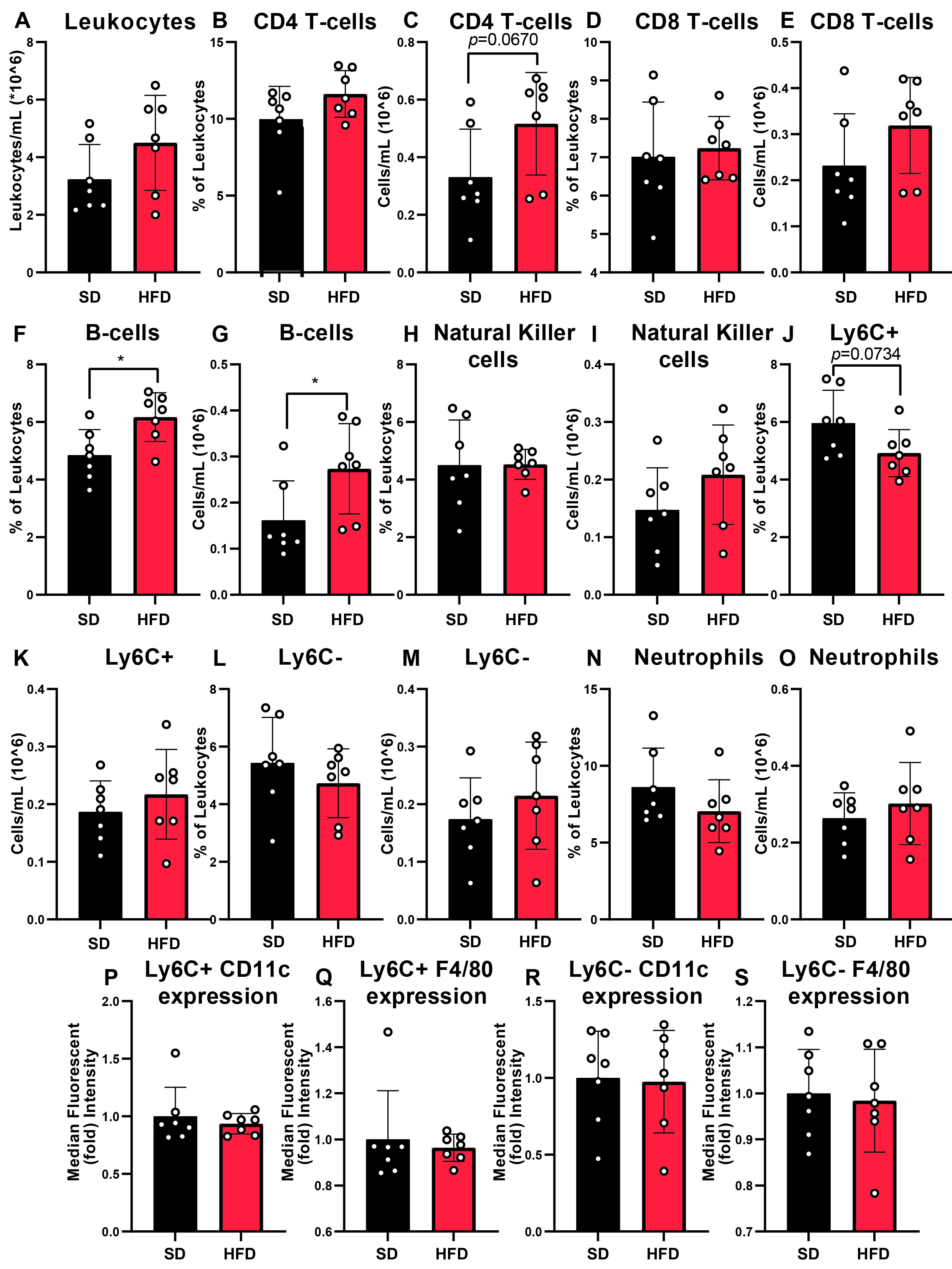

Supplement: Supplementary Figure 3 — Peripheral immunophenotyping by flow cytometry. Data represented as leukocytes (number of cells A), CD4 T-cells (% of cells or number of cells; B, C), CD8 T-cells (% of cells or number of cells; D, E), B-cells (% of cells or number of cells; F, G), natural killer cells (% of cells or number of cells; H, I), Ly6C+ monocytes (% of cells or number of cells; J, K), Ly6C- monocytes (% of cells or number of cells; L, M), neutrophils (% of cells or number of cells; N, O), Ly6C+ monocytes CD11c expression (median fluorescent (fold) intensity; P), Ly6C+ monocytes F4/80 expression (median fluorescent (fold) intensity; Q), Ly6C- monocytes CD11c expression (median fluorescent (fold) intensity; R), and Ly6C- monocytes F4/80 expression (median fluorescent (fold) intensity; S) in male BL6 mice fed high-fat diet (HFD) versus standard diet (SD); *p<0.05. [file Image_3.jpeg]

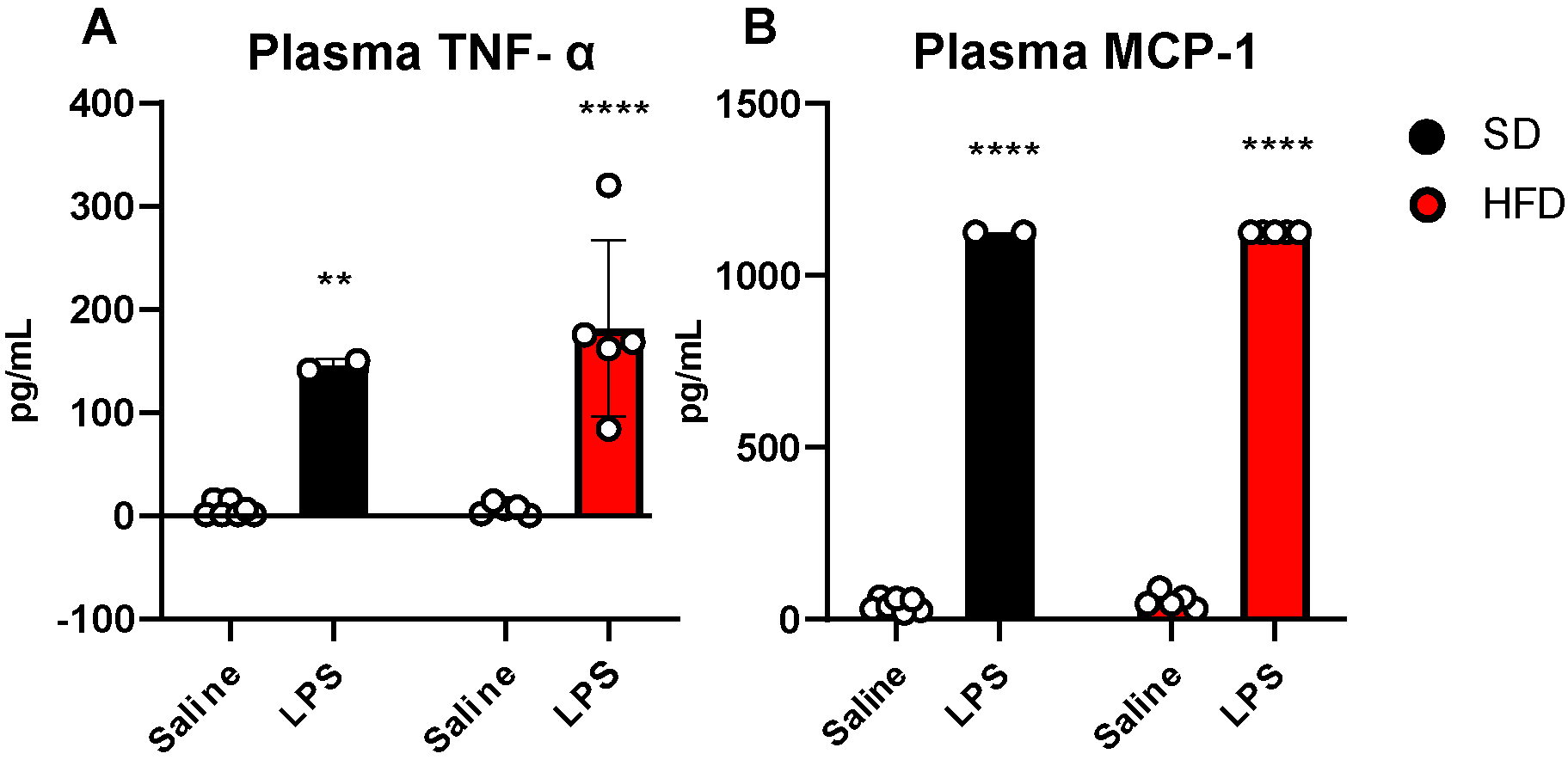

Supplement: Supplementary Figure 4 — Plasma inflammatory cytokines as measured by ELISA. Data represented as plasma TNF-α (pg/mL; A) and plasma MCP-1 (pg/mL; B) concentrations in male BL6 mice fed high-fat diet (HFD) versus standard diet (SD), administered either saline or lipopolysaccharide (LPS); **p<0.01, ****p<0.0001. [file Image_4.tif]

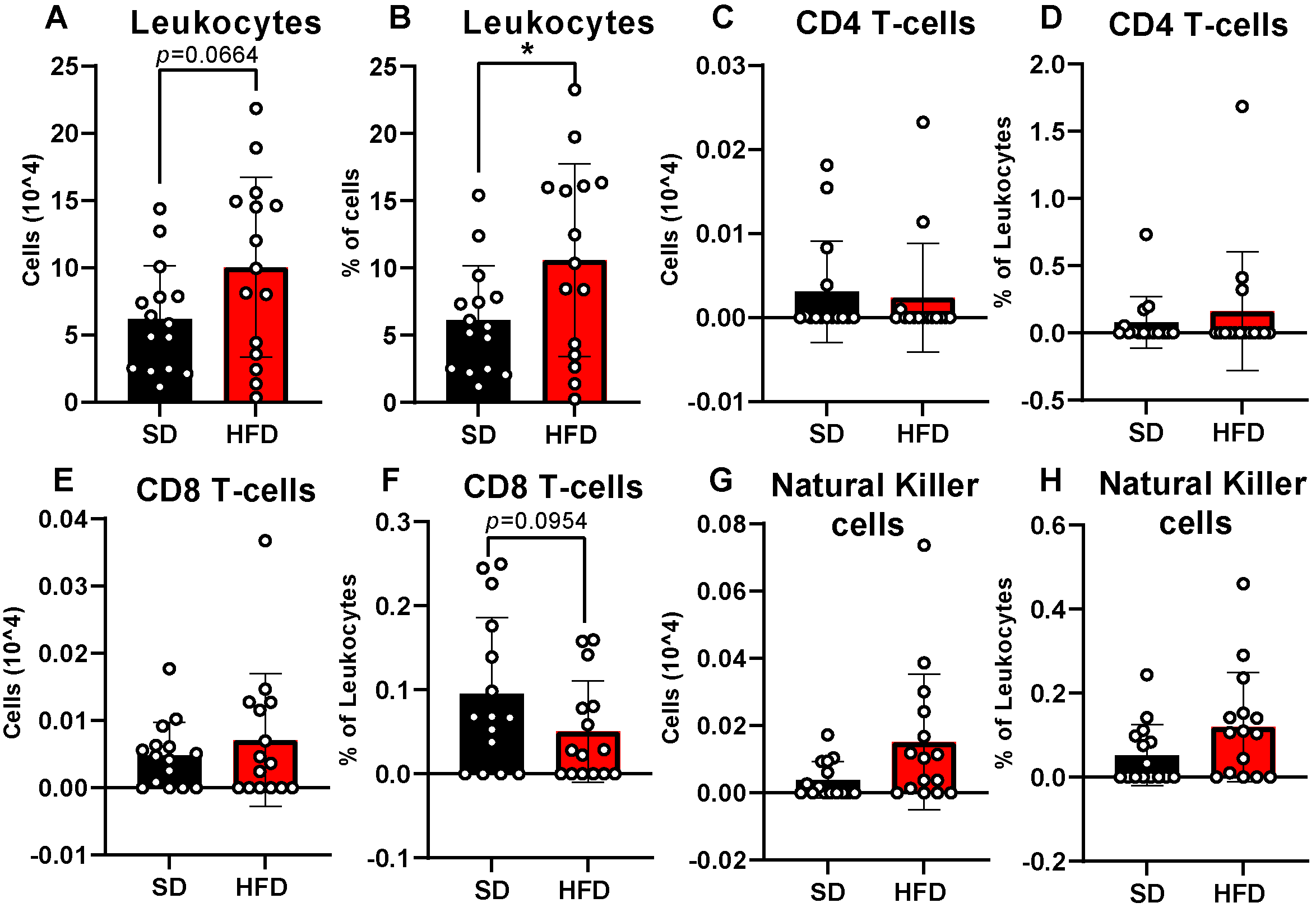

Supplement: Supplementary Figure 5 — CNS immunophenotyping of lymphoid cells by flow cytometry. Data represented as leukocytes (number of cells or % of cells; A, B), CD8 T-cells (number of cells or % of cells; C, D), CD4 T-cells (number of cells or % of cells; E, F), and natural killer cells (number of cells or % of cells; G, H) in male BL6 mice fed high-fat diet (HFD) versus standard diet (SD). There were no differences between animals administered saline vs. lipopolysaccharide (LPS), therefore SD and LPS animals were combined within their appropriate dietary groups; *p<0.05. [file Image_5.tif]

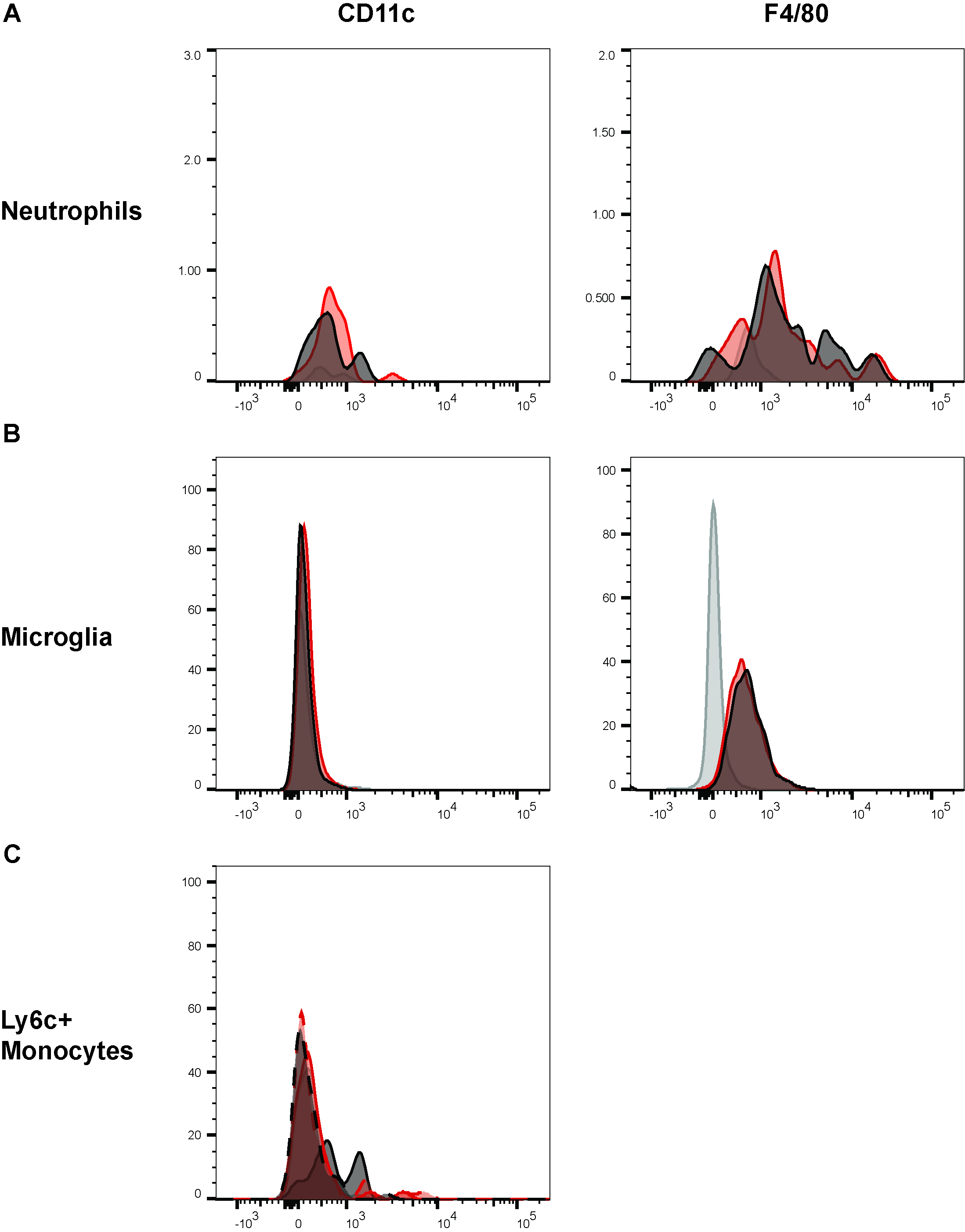

Supplement: Supplementary Figure 6 — Representative flow cytometry panels for CNS myeloid cells. Data represented as CD11c or F4/80 surface expression on neutrophils (A), microglia (B), and monocytes (C) in male BL6 mice fed high-fat diet (HFD; black) versus standard diet (SD; red) given saline. For monocytes, solid lines represent mice given saline and dashed lines represent mice given lipopolysaccharide. Grey peaks represent IgG control antibody. [file Image_6.tif]

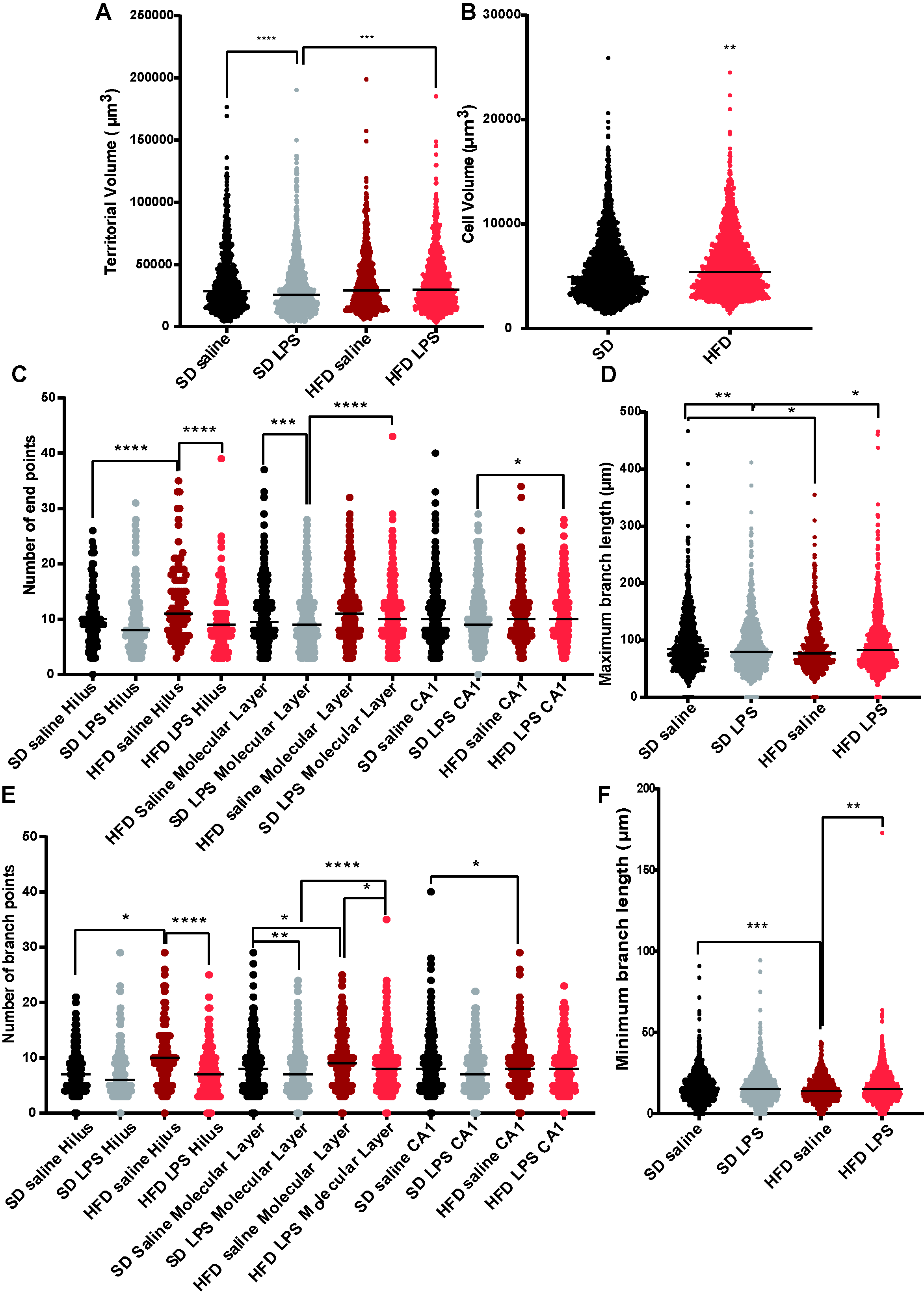

Supplement: Supplementary Figure 7 — Microglial morphology. Quantification of microglial territorial volume (μm3; A), cell volume (μm3; B), number of end points (C), maximum branch length (μm; D), number of end points (E), and minimum branch length (μm; F) in male BL6 mice fed high-fat diet (HFD) versus standard diet (SD) administered saline or LPS (lipopolysaccharide; A-D). Quantification of microglia percentage occupied volume (E) and average branch length (F). For A, B, D, and F quantification was performed on individual cells per image (n=3 images for the CA1 and molecular layers of the hippocampus and n=2 images for the hilus). For C and E quantification was performed by combing all images per hippocampal region. In the absence of differences between saline and LPS, data for each dietary group were combined and are presented as SD vs. HFD alone; *p<0.05, **p<0.01, *** p<0.001, ****p<0.0001. [file Image_7.tif]

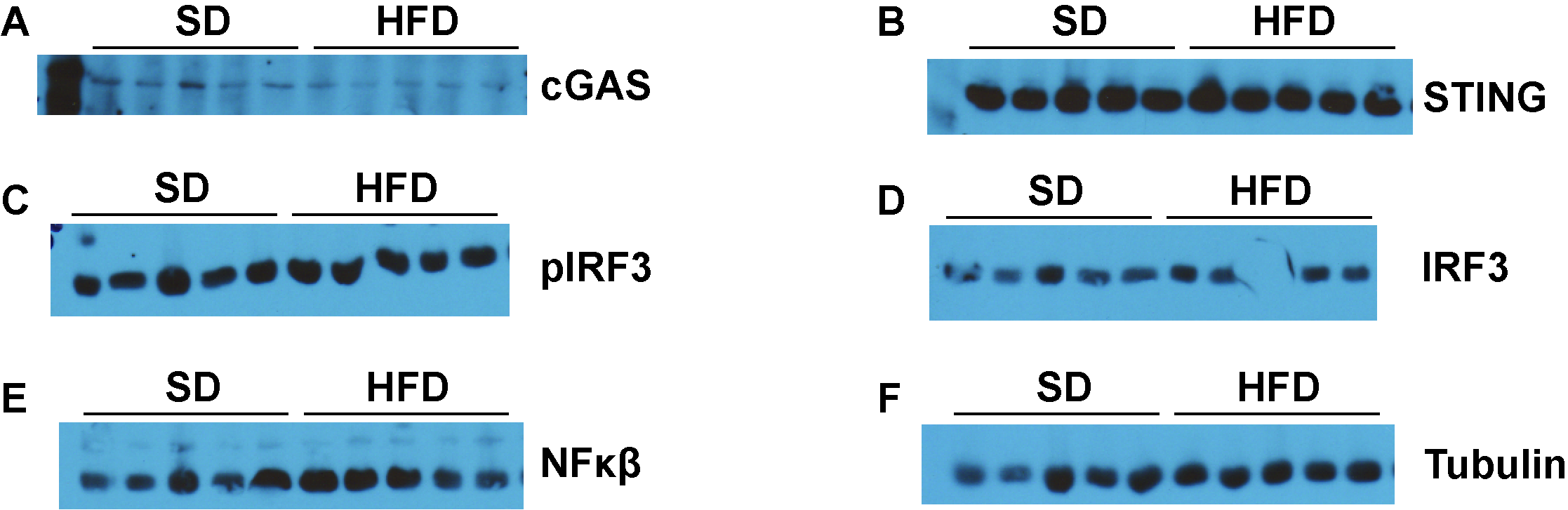

Supplement: Supplementary Figure 8 — Representative hippocampal cGAS/STING western blot images. Representative unaltered images of western blots quantified in main figure 4 (w/link color) for hippocampal cGAS/STING pathway protein expression of cGAS (A), STING (B), pIRF3 (C), IRF3 (D), NFκβ (E), and tubulin (F) in male BL/6 mice fed standard diet (SD) or high fat diet (HFD). [file Image_8.tif]

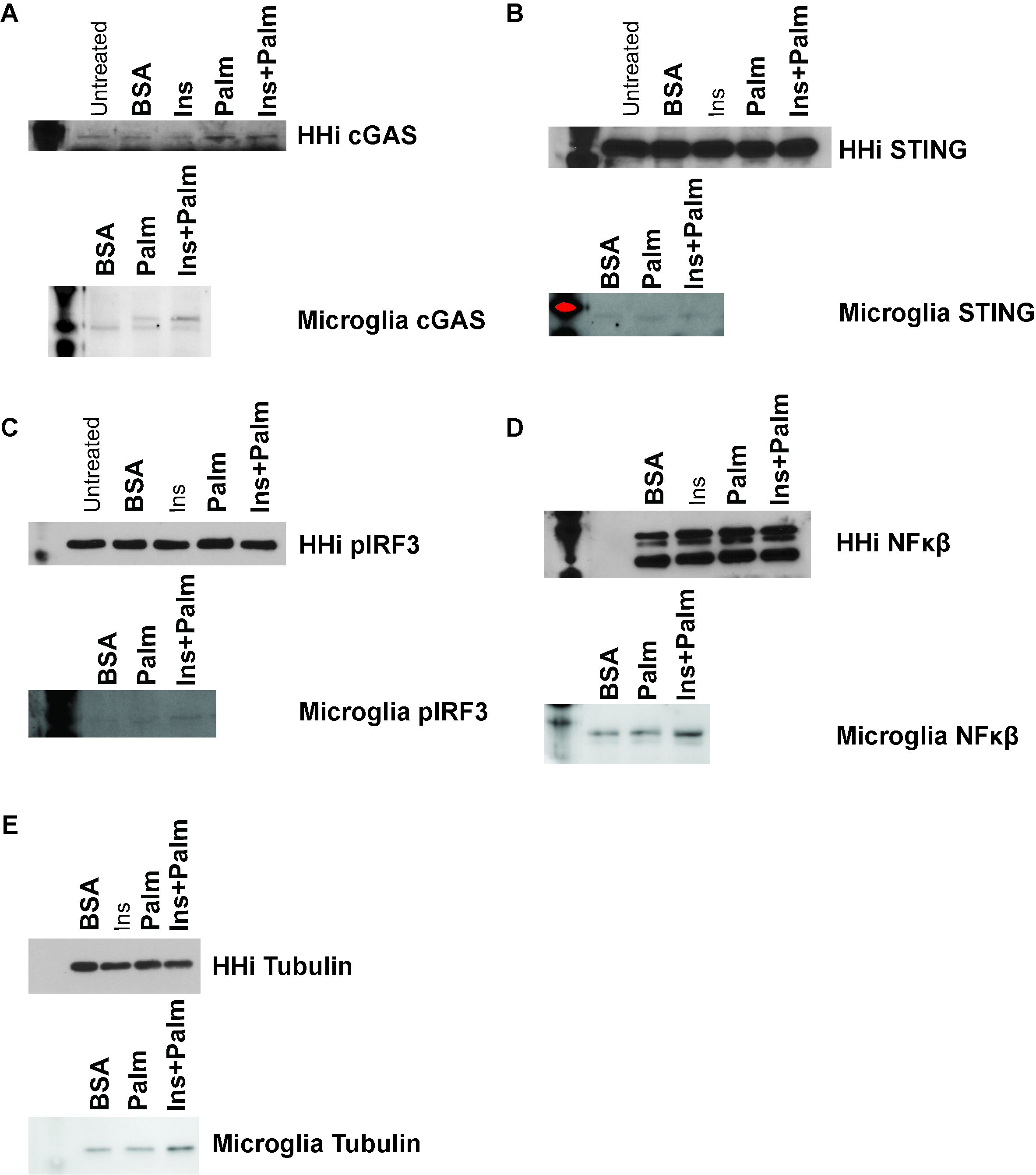

Supplement: Supplementary Figure 9 — Representative neuron and microglia cGAS/STING western blot images. Representative unaltered images of western blots quantified in main figure 6 (w/link color) for cGAS/STING pathway protein expression of cGAS (A), STING (B), pIRF3 (C), NFκβ (D), and tubulin (F) in palmitate (Palm) and insulin (Ins) stimulated neuronal and microglial cell lines. Conditions in bold are those used for analysis. [file Image_9.tif]

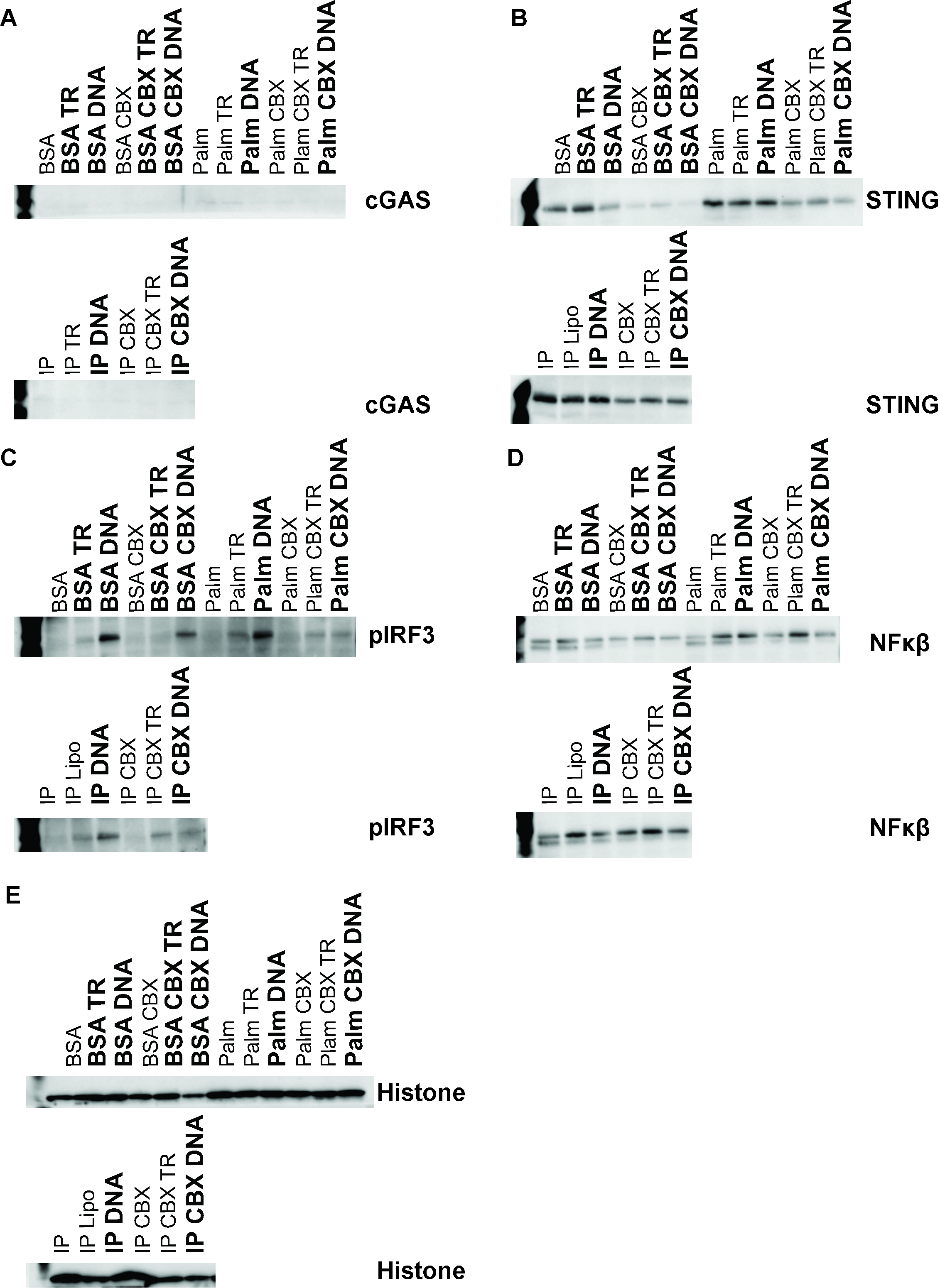

Supplement: Supplementary Figure 10 — Representative co-culture cGAS/STING western blot images. Representative unaltered images of western blots quantified in main figure 7 (w/link color) for cGAS/STING pathway protein expression of cGAS (A), STING (B), pIRF3 (C), NFκβ (D), and histone (F) in in palmitate (Palm) and insulin and palmitate (IP) stimulated neuronal and microglial cell line co-culture +/- the gap junction inhibitor carbenoxolone (CBX; 150 μM). Co-cultures were further stimulated with the dsDNA analog Poly dA:dT (DNA; 1μg/mL). Conditions in bold are those used for analysis. TR; transfection reagent. [file Image_10.tif]
